# Genetic blueprint of herbaceous peony floral scent: evidence from terpene synthase, Nudix hydrolase, and prenyltransferase

**DOI:** 10.1093/hr/uhag091

**Published:** 2026-03-09

**Authors:** Tingting Bao, Kimani Shadrack, Xiaotong Shan, Hongjie Li, Luhong Leng, Yueqing Li, Zhiqiang Wu, Xiang Gao

**Affiliations:** State Key Laboratory of Tropical Crop Breeding, Shenzhen Branch, Guangdong Laboratory of Lingnan Modern Agriculture, Key Laboratory of Synthetic Biology, Ministry of Agriculture and Rural Affairs, Agricultural Genomics Institute at Shenzhen, Chinese Academy of Agricultural Sciences, Shenzhen 518120, China; Key Laboratory of Molecular Epigenetics of MOE and Institute of Genetics & Cytology, Northeast Normal University, Changchun 130024, China; School of Pure and Applied Sciences, Karatina University, 1957-10101 Karatina, Kenya; Key Laboratory of Molecular Epigenetics of MOE and Institute of Genetics & Cytology, Northeast Normal University, Changchun 130024, China; Key Laboratory of Molecular Epigenetics of MOE and Institute of Genetics & Cytology, Northeast Normal University, Changchun 130024, China; Key Laboratory of Molecular Epigenetics of MOE and Institute of Genetics & Cytology, Northeast Normal University, Changchun 130024, China; Key Laboratory of Molecular Epigenetics of MOE and Institute of Genetics & Cytology, Northeast Normal University, Changchun 130024, China; State Key Laboratory of Tropical Crop Breeding, Shenzhen Branch, Guangdong Laboratory of Lingnan Modern Agriculture, Key Laboratory of Synthetic Biology, Ministry of Agriculture and Rural Affairs, Agricultural Genomics Institute at Shenzhen, Chinese Academy of Agricultural Sciences, Shenzhen 518120, China; Key Laboratory of Molecular Epigenetics of MOE and Institute of Genetics & Cytology, Northeast Normal University, Changchun 130024, China

## Abstract

Volatile terpenes constitute a predominant class of floral scent emitted by *Paeonia lactiflora.* Despite their ecological and economical significance, the genetic blueprint of the underlying biosynthetic pathway remains poorly elucidated. Although a few terpene synthase (*TPS*) genes have been reported, the broader network of genes orchestrating terpene production in *P. lactiflora* is still largely unresolved. In this study, we attempted to address this gap by exploring the terpene biosynthetic pathway genes in *P. lactiflora* ‘Zifengyu’. β-caryophyllene, geraniol, citronellol, and 1, 8-cineole were identified as the dominant floral terpenes, and catalytic functions of key proteins—terpene synthase (PlTPS), Nudix hydrolase (PlNUDX), and prenyltransferase (PlPT) were comprehensively characterized. Briefly, biochemical analyses revealed that six of the nine identified PlTPS proteins utilized diverse prenyl diphosphates to generate both monoterpenes and sesquiterpenes, while their products specificity were determined by plastidic or cytosolic localizations *in planta*. In particular, PlTPS4, PlTPS5, and PlTPS9 catalyzed the production of β-caryophyllene, 1, 8-cineole, and geraniol, respectively. Besides, two amino acid residues were found to drive catalytic activity and product profiles in PlTPS4 and PlTPS5. Markedly, PlNUDX hydrolyzed GPP and NPP to yield geraniol and nerol thereby providing a plastid-independent pathway for monoterpene biosynthesis, and prenyltransferases were further functionally characterized to clarify the supply of prenyl diphosphates feeding into volatile terpenes. Collectively, these findings not only provide a mechanistic framework for understanding floral terpene biosynthesis in *P. lactiflora* but also reveal alternative metabolic routes that enrich its volatile profiles that could be utilized in scent improvement of ornamental plants.

## Introduction

Floral scent, a complex blend of volatile organic compounds (VOCs), is a multifunctional plant trait that mediates critical ecological interactions, including pollinator attraction, herbivore defense, and plant communication, while driving the ornamental and commercial value of horticultural crops [1–3]. Horticulturally, floral scent is a key target for breeding programs, as scented cultivars command higher market demand for cut flowers, potted plants, and essential oil production. Among floral VOCs, volatile terpenes including monoterpenes (C_10_) and sesquiterpenes (C_15_) represent the largest and most functionally diverse class.

Terpene biosynthesis in plants relies on two conserved pathways: the cytosolic mevalonate (MVA) and plastidic methylerythritol phosphate (MEP) pathways, which produce the universal precursors isopentenyl diphosphate (IPP) and dimethylallyl diphosphate (DMAPP) [1, 4]. The structural diversity of floral terpenes is primarily shaped by three key enzyme families: prenyltransferases (PTs)**,** which condense IPP and DMAPP into intermediates like geranyl diphosphate (GPP, C_10_), and farnesyl diphosphate (FPP, C_15_) [5–9]; terpene synthases (TPSs), which generate core terpene skeletons from these intermediates [10–17], and Nudix hydrolases (NUDXs), which provide a TPS-independent route to produce terpene alcohols such as geraniol and farnesol [8, 18–20]. While canonical TPS pathways are well characterized in many plants, the coordination of *PT*, *TPS*, and *NUDX* gene families in shaping tissue- and stage-specific scent profiles remains poorly understood, particularly in nonmodel ornamental species.

The TPSs are a diverse group of enzymes that catalyze the formation of terpenes with varying chain lengths, including monoterpenes (C_10_), sesquiterpenes (C_15_), and diterpenes (C_20_), from substrates such as GPP, FPP, and GGPP [17, 21]. Most TPSs are involved in the biosynthesis of specialized metabolites crucial for ecological interactions, while a few contribute to primary metabolism like gibberellin production. The TPS enzymes possess two conserved domains: the N-terminal domain (PF01397) with the RR(x)_8_W motif and the C-terminal domain (PF03936) containing the DDxxD and NSE/DTE motifs, essential for metal ion coordination and active site stability [22]. Structural studies reveal that plant TPSs are broadly classified into at least eight subfamilies (TPS-a to TPS-h) based on lineage distribution, sequence homology, and function ([11]; Zhou et al., 2020a). Generally, TPS-a encodes sesquiterpene synthases, TPS-b and TPS-g are angiosperm-specific mono-TPSs with TPS-g lacking the RR(x)_8_W motif, and TPS-e/f subfamily members are involved in gibberellin biosynthesis. This structural and phylogenetic diversity underlies the functional specialization of TPSs across plant lineages. However, the broad substrate promiscuity and products infidelity of TPSs have been extensively reported, rendering their exact functional assignments inherently unpredictable [23, 24]. Besides, the TPS enzymes share a common fold comprised of variations of three conserved helical domains: α, βα, or γβα [25, 26]. Functional differences within these domains distinguish two major classes of TPSs: class II TPSs generate the initial carbocation intermediate via substrate protonation and catalyze scaffold rearrangements without cleaving the diphosphate ester bond, whereas class I TPSs use ionization of the diphosphate moiety to form the intermediary carbocation [27, 28]. Subsequently, a series of carbon skeleton rearrangements, methyl shifts, and hydride transfers are orchestrated, culminating in a wide array of terpene structures [30, 87]. The complicated catalytic properties of TPS proteins contribute to the vast diversity of volatile terpenes released from angiosperm plants, whereas the underlying mechanism is still obscure.

NUDX plays an important role in information transmission, plant growth coordination, and responses to adverse stresses [31–33]. Members of the NUDX family share a highly conserved amino acid sequence known as the NUDX box (Gx_5_Ex_7_REUxEExGU), where U represents a large hydrophobic amino acid [34, 35]. These enzymes primarily hydrolyze primary metabolites including nucleoside diphosphates, nucleoside triphosphates, coenzyme A, NAD(P)H, and capped mRNAs [36]. In recent years, NUDXs have also been implicated in plant terpenoid metabolism, e.g. catalyzing the hydrolysis of GPP, IPP, DMAPP, and FPP to generate the corresponding monophosphate products [18–20]. Notably, the Nudix hydrolase RhNUDX1 (*Rosa × hybrida*) converts GPP to geranyl phosphate (GP), which is subsequently hydrolyzed to geraniol by a phosphatase [8, 19]. The *NUDX* family genes have been well studied in plants such as *Arabidopsis* [36], barley [37], *Rosa* [20, 38], *Camellia* [39], and *Musa* spp [40]. However, the contribution of NUDX-mediated pathway to volatile terpene biosynthesis needs to be further evaluated in more plant species.

To supply the precursors of terpenoids, IPP and DMAPP, synthesized via the MEP and MVA pathways, are condensed by a class of short-chain trans-PTs via head-to-tail coupling, and the condensation results in the formation of GPP, FPP, and GGPP *etc*. For a long time, the prevailing view held that GPP and GGPP are biosynthesized in plastids from MEP pathway-derived IPP/DMAPP by GPP synthases (GPPSs) and GGPP synthases (GGPPSs), respectively. In contrast, FPP was thought to be produced in the cytosol and mitochondria from MVA pathway-derived precursors via FPP synthases (FPPSs). Among plant short-chain trans-PTs, all are homomeric enzymes with the exception of GPPSs, which can exist as either homomeric or heteromeric complexes. Homomeric GPPS complexes have been identified in gymnosperms and certain angiosperm species [41–44], whereas heteromeric GPPSs have been documented exclusively in angiosperms. Heteromeric GPPS complexes are composed of two subunits: a large subunit (GPPS-LSU) and a small subunit (GPPS-SSU). The GPPS-LSU is structurally related to or identical to GGPPSs [45–47] and exhibits inherent GGPPS activity when present alone. In contrast, the GPPS–SSU shares ~20% sequence similarity with plant GGPPSs or GPPSs and is generally devoid of catalytic activity [48]. Typically, two distinct types of SSUs have been characterized: SSU-I and SSU-II. Interaction between SSU-I and its respective large subunit seems to alter the latter’s product specificity, shifting it toward GPP production [42, 45–47, 49–52]. In contrast, SSU-IIs perform two main functions: they either boost the activity and product specificity of GGPPSs ([47]; Zhou et al., 2020b), or they promote the channeling of GGPP to specific downstream metabolic pathways. However, the aforementioned ‘classical’ perspective has changed in recent years due to the multiple subcellular localizations of GGPPSs and the presence of a GPP pool in the cytosol. For example, GGPPSs are present not only in their typical plastidial location but also in mitochondria and the endoplasmic reticulum (ER) [53, 54]. In addition, PTs capable of producing GPP in the cytosol were recently isolated and characterized from *Lithospermum erythrorhizon* [55, 56], roses [7], and pelargonium [57]. However, how PTs contribute to the cytosolic GPP pool in other plant species is largely unknown.

Within the Paeonia genus, both herbaceous peonies (*Paeonia lactiflora*) and tree peonies (*P. suffruticosa*) are globally renowned for their ornamental value, with their scent profiles exhibiting complex blends of terpenes, benzenoids, and fatty acid derivatives [58–61]. *P. lactiflora*, in particular, holds immense cultural and economic significance as a cut flower and garden plant, yet our understanding of its scent biosynthesis remains fragmented. Previous studies have identified several *TPS* genes in specific cultivars [60, 62, 63], but the complete network—including the coordination of PTs that supply precursor molecules, alternative TPS-independent pathways (e.g. Nudix hydrolases), and the regulatory mechanisms shaping tissue- and stage-specific scent emission—remains largely unexplored. This knowledge gap limits targeted breeding efforts to enhance or diversify scent traits in *P. lactiflora*, underscoring the need for a comprehensive analysis of its terpene biosynthesis.

In the present study, we employed *P. lactiflora* ‘Zifengyu’, a cultivar renowned for its distinctive floral fragrance, as a representative system to unravel the molecular basis of scent formation. Volatile profiling revealed that β-caryophyllene was the dominant sesquiterpene, accompanied by monoterpenes including geraniol, citronellol, 1, 8-cineole, suggesting an active and metabolically diverse terpenoid biosynthetic network. To dissect the genetic and biochemical underpinnings of this diversity, we integrated expression profiling, evolutionary relationships, subcellular localization, and both *in vitro* and *in planta* enzymatic assays of TPS, NUDX, and PT. This multifaceted approach uncovered the coordinated action of these three gene families in shaping the floral volatile profile of *P. lactiflora.* Beyond elucidating a mechanistic framework for terpene biosynthesis in peony, our findings highlight novel enzymatic routes and regulatory nodes that can be leveraged as genetic targets for the enhancement of floral fragrance in ornamental breeding.

## Results

### Volatile terpene analysis reveals β-caryophyllene, citronellol, (*Z*)-β-ocimene, 1, 8-cineole, and geraniol as key floral scent compounds in *P. lactiflora* ‘Zifengyu’

**Figure 1:**
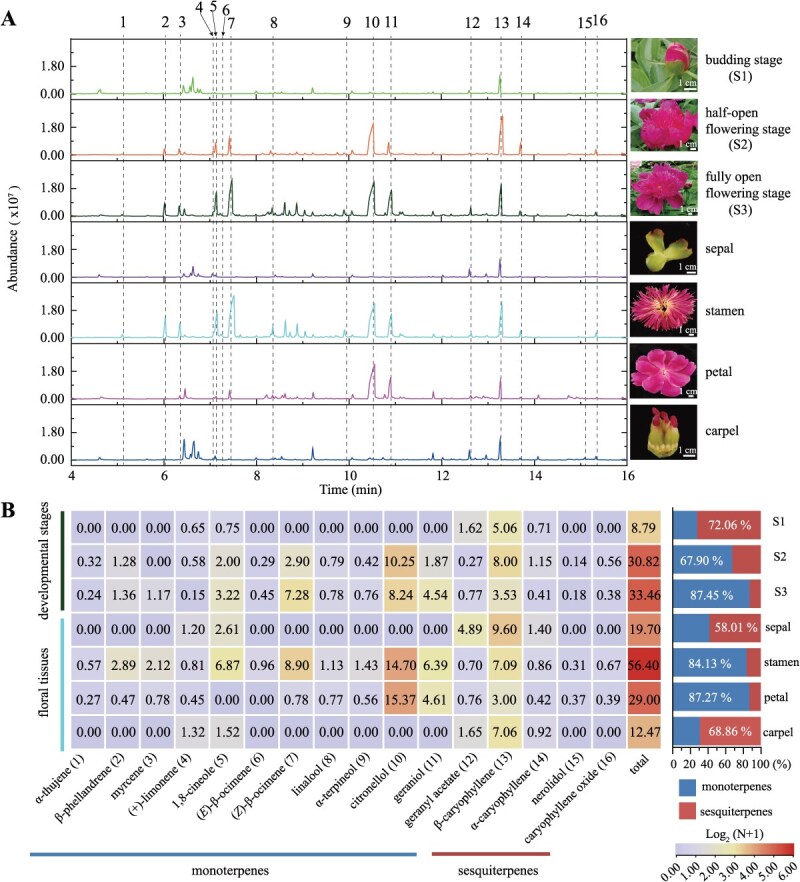
The volatile terpene release pattern from the flowers of *P. lactiflora* ‘Zifengyu’. **(A)** The total ion chromatogram of volatile terpenes released from flowers at different developmental stages and tissues from fully opened flowers. **(B)** Relative release of different volatile terpenes from flowers at different developmental stages and tissues from fully opened flowers. The volatile terpenes were analyzed by HS-SPME-GC–MS. Compound identification was performed by comparing spectra with those in the NIST-2017 mass spectral library. The relative release was calculated relative to standard β-caryophyllene [10] and the unit of content was ng/g fresh weight.

To characterize the VOC profiles of *P. lactiflora* ‘Zifengyu’ flowers, we performed headspace solid-phase microextraction-gas chromatography-mass spectrometry (HS-SPME-GC–MS) analysis. As narrated in our previous studies [58], terpenoids appeared as leading VOCs, followed by volatile phenylpropanoid-related (C6-C2) compounds, and fatty acid derivatives in *P. lactiflora* ‘Zifengyu’. To better define the volatile terpene emission pattern, we categorized floral development into three stages (Fig. 1A): flower bud (S1), half-open flower (S2), and fully open flower (S3). Volatile analysis showed that total volatile terpene emission increased during flower development, primarily driven by elevated monoterpene release (Fig. 1B, Supplementary Table S1). Specifically, β-caryophyllene, one of the few sesquiterpenes detected, was the dominant volatile emission at the bud stage, peaked at the half-opened stage, and subsequently declined (Fig. 1B). As flower matured, volatile monoterpenes including citronellol, (*Z*)-β-ocimene, 1, 8-cineole, and geraniol were released in large quantities, suggesting uncharacterized but potentially pollinator-attracting roles (Fig. 1B).

To associate specific floral tissues with their volatile emission profiles, we analyzed VOCs from sepals, stamens, petals, and carpels of fully open flowers. β-caryophyllene was detected across all four tissues, showing comparable emission levels. In contrast, citronellol and geraniol were exclusively emitted from petals and stamens, while (*Z*)-β-ocimene and 1, 8-cineole were primarily released from stamens (Fig. 1B). Additionally, monoterpene had overwhelming release levels when compared with volatile sesquiterpenes in stamens and petals. Biologically, this tissue-specific distribution suggests reprogramming of terpene biosynthesis during flower maturation, aligning with the plant’s reproductive strategy to enhance pollinator attraction at peak bloom. However, the specific biological functions of these volatile terpenes in individual tissues remain unclear.

### Tissue- and stage-specific expression reveals carpel-enriched, developmentally regulated C10/C15 terpene biosynthesis in *P. lactiflora* ‘Zifengyu’

**Figure 2:**
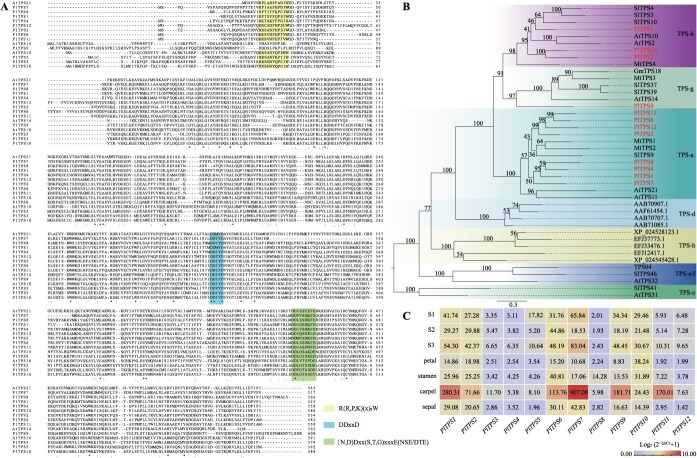
Screening and functional characterization of *TPS* genes in the *P. lactiflora* ‘Zifengyu’. **(A)** Sequence alignment of the 12 TPSs screened in *P. lactiflora* ‘Zifengyu’. The conserved motifs R(R,P,K)(x)_8_W, DDxx(D/E), and (N,D)Dxx(S,T,G)xxxE (NSE/DTE) were highlighted with different backgrounds. **(B)** Maximum likelihood tree (MLtree) of TPS proteins from *P. lactiflora* ‘Zifengyu’ and other plants. Numbers on branches indicate percentage of replicate trees in which the associated taxa clustered together in the bootstrap test (1000 replicates). The tree was drawn to scale, with branch lengths in the same units as those of the evolutionary distances used to infer the phylogenetic tree. The evolutionary distances were computed in the units of the number of amino acid substitutions per site, as shown by the scale below the tree. GenBank accessions of other TPS proteins were provided in Supplementary Table S2. The phylogenetic tree is constructed through TBtools II software [67]. **(C)** Relative expression levels of *TPS* genes in different tissues and developmental stages of peony. Relative quantitative gene expression was calculated using the 2^−ΔΔCт^ formula. The mean data were normalized by log_2_.

To identify putative *TPS* genes, our previous work annotated and assembled transcripts from *P. lactiflora* ‘Zifengyu’ floral database [58]. A total of 12 *TPS* genes were retrieved from the transcriptome database, among which *PlTPS2*, *PlTPS3*, *PlTPS5*, *PlTPS6*, *PlTPS8*, and *PlTPS11* showed high similarity to previously described terpene synthase genes (Supplementary Figs S1–S9, [58, 64–66]). BLAST alignment of amino acid sequence presented salient TPS features such as the presence of R(R,P,K)(x)_8_W, DDxx(D/E), and (N,D)Dxx(S,T,G)xxxE (NSE/DTE) motifs involved in catalysis (Fig. 2A). While, phylogenetic reconstruction grouped these peony TPS proteins into three major clades: TPS-a (TPS1, TPS2, TPS4, TPS6, TPS7, TPS8, TPS11, and TPS12), TPS-b (TPS3, TPS5, and TPS10), and TPS-g (TPS9), consistent with previous functional annotations in other plants (Fig. 2B; Supplementary Table S2). These clade classifications suggest functional conservation and potential diversification roles in terpenoid biosynthesis across tissues and developmental stages.

To evaluate the expression profile of the *PlTPS* genes in different tissues and flower development, the expression levels of the twelve *TPS* genes revealed spatial and temporal specificity (Fig. 2C, Supplementary Table S3). Specifically, *TPS1*, *TPS2*, *TPS6*, *TPS7*, *TPS9*, and *TPS11* were highly expressed in the carpel, exhibiting likelihood of these genes participating in reproductive development, defense, and scent-mediated interactions. Whereas, other *TPS* genes were moderately expressed in other floral organs, suggesting broader tissue functionality. Generally, *TPS1*, *TPS2*, *TPS6*, *TPS7*, *TPS9*, and *TPS10* were constantly expressed across all three flower developmental stages with higher expression levels (S1–S3), suggesting their involvement in the dynamic regulation of terpenoid production during floral maturation. In contrast, *TPS3*, *TPS4*, *TPS8*, *TPS11*, and *TPS12* showed low expression across all tissues and stages, implying limited or other specialized roles. Overall, tissue- and development-specific expression profile reveals carpel-enriched and developmentally regulated volatile terpenoid biosynthesis in *P. lactiflora* ‘Zifengyu’.

### *In vitro* biochemical analysis reveals the substrate promiscuity and products infidelity of TPS proteins

**Figure 3:**
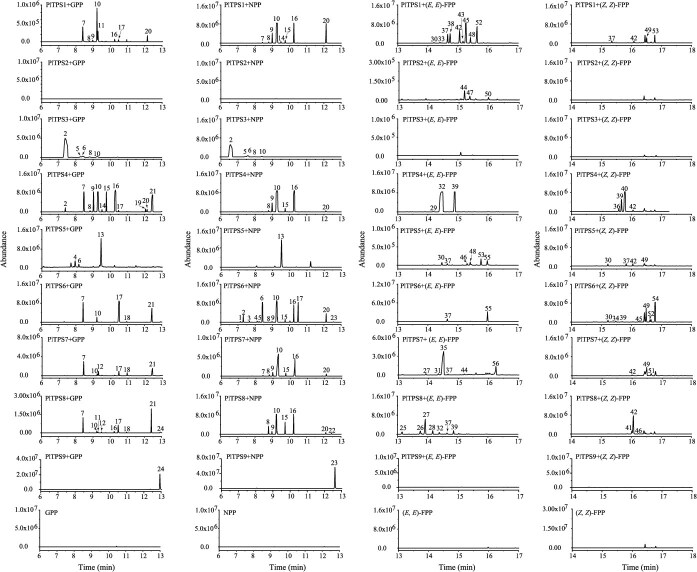
*In vitro* enzyme activity assay of peony TPS protein. The *x*-axis represents the retention time of the peak outflow, and the *y*-axis represents the integral area of the chromatographic peak. The volatiles were analyzed by HS-SPME-GC–MS. Compound identification was performed by comparing spectra with those in the NIST-2017 mass spectral library. The relative content of enzyme products was detailed in Supplemental Table S4. Numbers near the peaks represent different volatiles: 1, α-thujene; 2, α-pinene; 3, camphene; 4, sabinene; 5, (*1S*)-(−)-β-pinene; 6, β-pinene; 7, myrcene; 8, α-phellandrene; 9, α-terpinene; 10, limonene; 11, (−)-α-pinene; 12, (*E*)-β-ocimene; 13, 1, 8-cineole; 14, (*Z*)-β-ocimene; 15, γ-terpinene; 16, terpinolene; 17, linalool; 18, linalool methyl ether; 19, nerol methyl ether analog; 20, α-terpineol; 21, nerol methyl ether; 22, α-terpinyl acetate; 23, nerol; 24, geraniol; 25, β-cadinene; 26, (−)-α-gurjunene; 27, β-elemene; 28, α-guaiene; 29, isocaryophyllene; 30, α-bergamotene; 31, (−)-γ-elemene; 32, β-caryophyllene; 33, santalene; 34, α-bergamotene; 35, γ-elemene; 36, (+)-γ-gurjunene; 37, (*E*)-β-farnesene; 38, α-longipinene; 39, α-caryophyllene; 40, β-humulene; 41, (−)-α-acoradiene; 42, himachalene; 43, (*E*)-β-farnesene; 44, β-cubebene; 45, zingiberene; 46, α-farnesene; 47, (+)-ledene; 48, β-bisabolene; 49, α-longipinene; 50, (+)-δ-cadinene; 51, α-patchoulene; 52, β-sesquiphellandrene; 53, (*Z*)-α-bisabolene; 54, α-himachalene; 55, (*E*)-nerolidol；56, γ-elemene analog.

To investigate the enzymatic properties of TPSs in *P. lactiflora* ‘Zifengyu’, *in vitro* biochemical analyses of the purified recombinant proteins were performed, results demonstrated the substrate promiscuity and product profiles of various PlTPSs using four key prenyl diphosphate substrates: GPP, NPP, (*E, E*)-FPP, and (*Z, Z*)-FPP. Gas chromatography–mass spectrometry (GC–MS) analysis showed that distinct PlTPS enzymes catalyzed the conversion of these substrates into diverse terpenoid products with varying efficiency and product complexity (Fig. 3; Supplementary Table S4). For instance, PlTPS1 produced both sesqui- and monoterpenes, including zingiberene (Peak 45, 91.69 ± 6.55 ng, 23.73%), himachalene (Peak 42, 77.15 ± 1 ng, 19.89%), β-sesquiphellandrene (Peak 52, 75.71 ± 6.08 ng, 19.51%), and α-longipinene (Peak 38, 72.85 ± 0.09 ng, 18.76%) with (*E*, *E*)-FPP as substrate, while incubation with GPP generated limonene (Peak 10, 3.62 ± 0.01 ng, 46.46%), myrcene (Peak 7, 2.54 ± 0.13 ng, 20.01%), and (−)-α-pinene (Peak 11, 2.37 ± 0.01 ng, 15.97%). PlTPS2 was only active with (*E*, *E*)-FPP, yielding minor sesquiterpenes such as β-cubebene (Peak 44, 2.23 ± 0.01 ng, 87.56%). PlTPS3 catalyzed the formation of α-pinene (Peak 2), β-pinene (Peak 6), and limonene (Peak 10) with GPP/NPP as substrates; PlTPS4 produced β-caryophyllene (Peak 32, 71.02 ± 0.22 ng, 71.69%) and α-caryophyllene (Peak 39, 28.67 ± 0.09 ng, 27.88%) when incubated with (*E*, *E*)-FPP. PlTPS5 showed broad activity across substrates, generating 1, 8-cineole (Peak 13, 56.45 ± 6.84 ng, 42.6%), sabinene (Peak 4, 39.36 ± 6.25 ng, 29.02%), and β-pinene (Peak 6, 16.45 ± 2.51 ng, 11.35%) as major products using GPP as substrate. PlTPS6–PlTPS8 also demonstrated substrate promiscuity: PlTPS6 catalyzed linalool (Peak 17, 49.69 ± 3.56 ng, 50.99%) and (*E*)-nerolidol (Peak 55, 6.28 ± 0.09 ng, 75.59%) as products; PlTPS7 produced γ-elemene (Peak 35, 21.83 ± 2.51 ng, 70.16%) as major products with (*E*, *E*)-FPP as substrate; PlTPS8 yielded β-cadinene (Peak 25, 4.03 ± 0.02 ng, 8.31%), α-guaiene (Peak 28, 4.78 ± 0.03 ng, 11.02%), and β-elemene (Peak 27, 16.43 ± 0.01 ng, 52.96%) using substrate for sesquiterpene biosynthesis. Notably, PlTPS9 exclusively converted GPP to geraniol (Peak 24, 2.35 ± 0.05 ng, 100%) and NPP to nerol (Peak 23, 1.87 ± 0.02 ng, 100%). Collectively, our *in vitro* enzymatic assays identified candidate key TPSs responsible for the major volatile terpenes emitted by *P. lactiflora* ‘Zifengyu’ flowers, including geraniol synthase (PlTPS9), β-caryophyllene synthase (PlTPS4), and 1, 8-cineole synthase (PlTPS5). However, their physiological roles *in planta* await further characterization. Moreover, PlTPS proteins from ‘Zifengyu’ exhibited amino acid polymorphisms relative to allelic variants in other peony accessions, which may lead to different product profiles for most enzymes except PlTPS3 and PlTPS9 (Supplementary Figs S1–S9). For instance, TPS5 from *P. lactiflora* ‘Zifengyu’ differs by only 11 amino acid residues from the allelic variant PP889953.1 in *P. lactiflora* ‘Wu Hua Long Yu’ (Supplementary Fig. S6), yet these subtle sequence changes suffice to generate entirely distinct products (Fig. 3 and Supplementary Fig. S1). This observation highlights the profound impact of allelic amino acid variations on TPS functional divergence, emphasizing their critical role in driving the diversity of terpene metabolic outputs in peonies.

### *In vivo* functional characterization and subcellular targeting define TPS product fidelity

**Figure 4:**
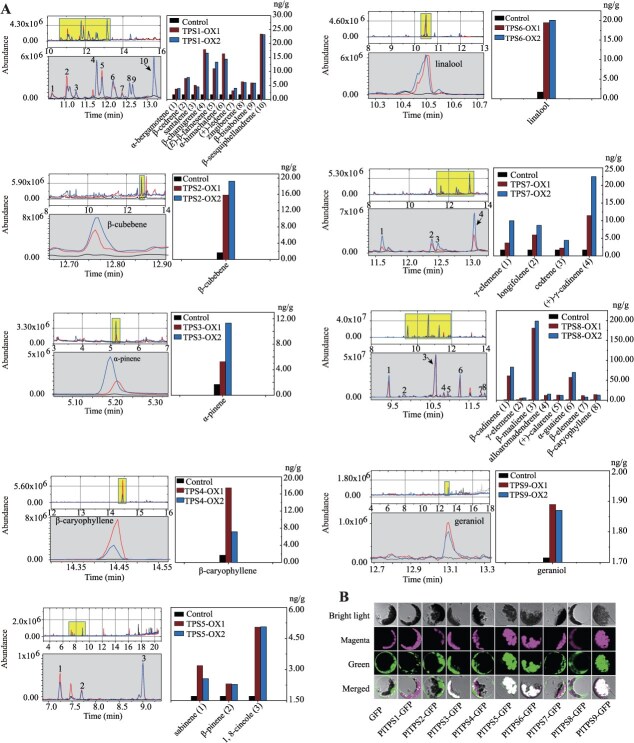
*In vivo* characterization of peony TPS protein by heterologous expression. **(A)** Types and contents of volatiles detected in the control and experimental groups after transient overexpression of *TPS* gene in *N. benthamiana* leaves. For each TPS, the upper left panel shows the ion peaks of the detected volatiles. The *x*-axis represents the retention time of the peak outflow, and the *y*-axis represents the integral area of the chromatographic peak. The highlighted part in the upper left panel was magnified and described in detail as the lower left panel. The right panel contains the relative contents of the detected volatile terpenes. The volatiles were analyzed by HS-SPME-GC–MS. Compound identification was performed by comparing spectra with those in the NIST-2017 mass spectral library. All the data were calculated relative to standard β-caryophyllene [10], and the unit was ng/g fresh weight. The relative contents of detected products were detailed in Supplemental Table S5. **(B)** Subcellular localization of peony TPS protein observed in *Arabidopsis* protoplasts. Bright light, bright field image; green, GFP fluorescence detected in the green channel; magenta, chlorophyll autofluorescence; merged, merged green and magenta channel images. Bars = 25 μm.

To further validate the functional roles of the nine highly expressed *TPS* genes aforementioned *in planta*, they were transiently overexpressed in *Nicotiana benthamiana* leaves under the control of the *35S* promoter. Volatile profiling of the infiltrated leaves revealed distinct terpene emission patterns (Fig. 4A; Supplementary Table S5). Notably, 35S::*PlTPS1* led to the production of β-sesquiphellandrene as the most abundant sesquiterpene, while 35S::*PlTPS2* exclusively accumulated β-cubebene. 35S::*PlTPS3* primarily emitted (*1R*)-(+)-α-pinene, whereas 35S::*PlTPS4* generated β-caryophyllene as the sole product. Similarly, 35S::*PlTPS5* yielded sabinene, β-pinene, and 1, 8-cineole (as the dominant product), while 35S::*PlTPS6* exclusively produced linalool. The 35S::*PlTPS7* line largely accumulated (+)-γ-cadinene, along with minor levels of longifolene, γ-elemene, and cedrene. 35S::*PlTPS8* was primarily responsible for the biosynthesis of sesquiterpenes including β-maaliene (190.00 ± 7.23 ng, 50.31%), β-cadinene (72.29 ± 8.92 ng, 18.46%), and α-guaiene (63.83 ± 4.92 ng, 17.71%) *etc*. 35S::*PlTPS9* exhibited strict product fidelity, synthesizing only geraniol, a key monoterpene alcohol. Overall, these *in vivo* results were largely consistent with the *in vitro* enzymatic assay findings (Fig. 3), confirming that PlTPSs are functionally active and capable of generating diverse monoterpene and sesquiterpene backbones. However, we were unable to identify any TPS enzymes responsible for the biosynthesis of β-ocimene and citronellol—two of the most abundant volatile compounds detected in *P. lactiflora* ‘Zifengyu’ flowers. Collectively, these findings underscore the functional diversity of peony TPS enzymes and highlight PlTPS4 and PlTPS5 as key players in the biosynthesis of signature floral volatiles, β-caryophyllene and 1,8-cineole, respectively.

Subcellular localization of PlTPSs revealed plastidic targeting of monoterpene-producing enzymes (PlTPS3, PlTPS5, PlTPS6, PlTPS9) and cytosolic localization of sesquiterpene-producing TPSs (PlTPS1, PlTPS2, PlTPS4, PlTPS7, PlTPS8) (Fig. 4B). These localization patterns were largely consistent with predictions generated by the TargetP-2.0 program (https://services.healthtech.dtu.dk/services/TargetP-2.0/) [29] (Supplementary Table S6), and aligned with MEP- and MVA-derived metabolic compartmentalization. Although TPS proteins featured substrate promiscuity, they could only generate monoterpenes or sesquiterpenes when overexpressed *in planta*, depending on their subcelluar localizations. When localized in plastid, these enzymes catalyze the formation of monoterpenes, whereas cytosolic localization directs the production of sesquiterpenes. Collectively, these *in vitro*, *in vivo*, and GFP-fusion localization data align closely with the terpenoid emission patterns observed across floral tissues and developmental stages in *P. lactiflora* (Fig. 1), reinforcing the biological significance of TPS enzyme functions in shaping terpene metabolic diversity.

### Active site residues determine product specificity of PlTPS4 and PlTPS5 in C10/C15 biosynthesis

The aforementioned characterizations identified PlTPS4 and PlTPS5 as key enzymes responsible for synthesizing the signature floral volatiles β-caryophyllene and 1,8-cineole, in *P. lactiflora* ‘Zifengyu’ flowers. To investigate how the activate sites of PlTPS4 and PlTPS5 interact with prenyl substrates to generate product diversity, we performed molecular docking of (*E, E*)-FPP and GPP into their respective models using ChimeraX/AutoDock Vina.

**Figure 5:**
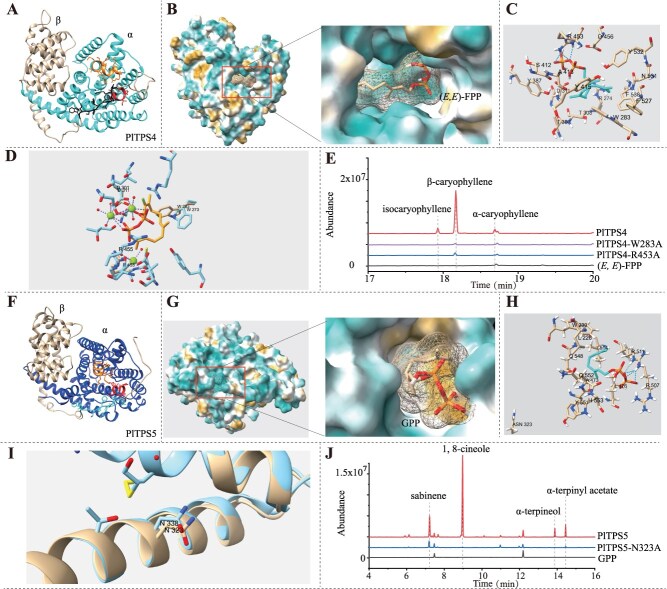
Screening of key amino acids that affect the function of TPS protein. **(A)** The structure model PlTPS4. The α, β fold are shown. The N-terminal domain (golden) and C-terminal domain (cyan) are shown. The DDxxD (red) and NSE/DTE (orange) are conserved motifs. **(B)** The left side is the hydrophobicity surface of PlTPS4. The right side is the topography of the PlTPS4 active site with docked (*E, E*)-FPP substrate (close view). **(C)** The structure of PlTPS-ligand complex, PlTPS4-FPP substrate complex. **(D)** Comparison of the structures of PlTPS4 with TEAS-resolved templates. Superposition of PlTPS4 with sesquiterpene TEAS template. Residues W283, D311, and R453 were aligned with TEAS residues W273, D301, and R455, respectively. **(E)**  *In vitro* enzyme activity assay of TPS4 protein and key amino acid site mutant proteins. **(F)** The structure model of PlTPS5. The α, β fold and C-terminal are shown. The N-terminal domain (golden) and C-terminal domain (blue) are shown. The RR(x)_8_W. (black) DDxxD (red) and NSE/DTE (orange). **(G)** The left side is the hydrophobicity surface of PlTPS5. The right side is the topography of the PlTPS5 active site with docked GPP substrate (close view). **(H)** The structure of PlTPS-ligand complex, PlTPS5-GPP substrate complex. **(I)** Superposition of PlTPS5 and monoterpene SfCS1 template. Asn-338 in SfCS1 is aligned with Asn-323 residue in TPS5. **(J)**  *In vitro* enzyme activity assay of TPS5 protein and mutant proteins at key amino acid sites. The volatiles were analyzed by HS-SPME-GC–MS. Compound identification was performed by comparing spectra with those in the NIST-2017 mass spectral library.

For PlTPS4, structural modeling confirmed the presence of canonical TPS motifs, including the metal-binding DDxxD and NSE/DTE triads, which coordinate divalent cations essential for catalytic activity (Fig. 5A). Further docking with (*E, E*)-FPP reveled the active cavity responsible for substrate binding and carbocation stabilization (Fig. 5B). To identify key residues adjacent to the active site, we analyzed atoms within 3 Å of the docked ligand, pinpointing 15 amino acid residues (Fig. 5C). Among these, D311 and D456 were located in the DDxxD and NSE/DTE domains, respectively. To validate the functional importance of predicted residues, we mutated asparagine (N283)and arginine (R453) to alanine (A) (Fig. 5D and E). Mutations at these sites resulted in a near-complete loss of β-caryophyllene synthase activity (Fig. 5E), and this functional impairment was recapitulated in transient expression assays in *N. benthamiana* leaves (Supplementary Fig. S10A).

Similarly, structural modeling and docking of PlTPS5 with GPP identified 12 amino acid residues within 3 Å of the substrate, of which only L476 resided in the NSE/DTE domain (Fig. 5F–H). To confirm these findings, we mutated asparagine (N323) in PlTPS5 to alanine (A), which abolished the enzyme’s ability to synthesize 1, 8-cineole (Fig. 5I and J; Supplementary Table S4). Transient expression of the *PlTPS5-N323A* mutant in *N. benthamiana* leaves further validated the result, as no 1,8-cineole was detected in transgenic tissues (Supplementary Fig. S10B; Supplementary Table S5). These observations suggest that PlTPS5 functions as a specialized 1,8-cineole synthase with partial product infidelity, enabling alternative carbocation rearrangements and hydration reactions. The high yield of 1,8-cineole implies efficient stabilization of the α-terpinyl cation intermediate, followed by intramolecular ether formation ([68]; Supplementary Fig. S11). Together, these biochemical and structural results define the functional specialization and critical catalytic residues of PlTPS4 and PlTPS5, highlighting their roles in shaping the volatile terpene profile of *P. lactiflora* ‘Zifengyu’.

### NUDX and PlTPS9 likely contribute to biosynthesis of geraniol and its derivatives in *P. lactiflora* ‘Zifengyu’

**Figure 6:**
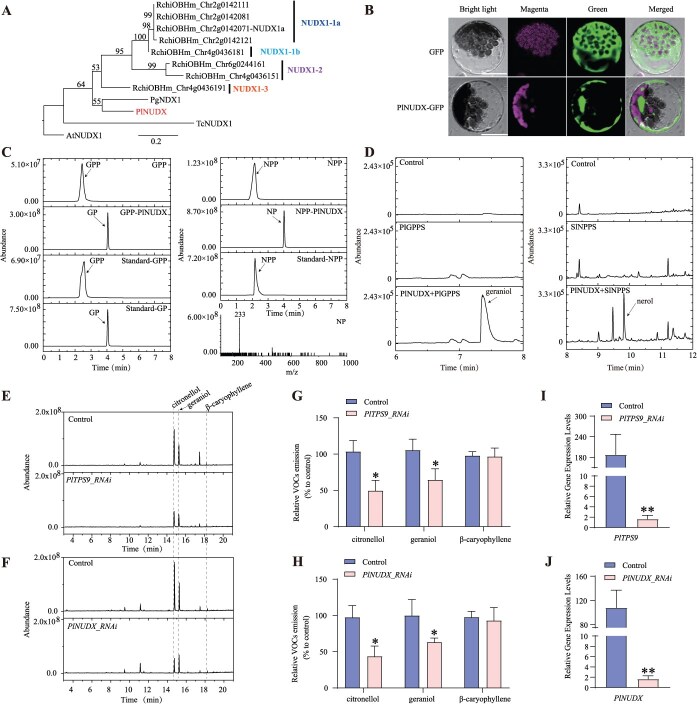
Functional identification of NUDX protein from peony. **(A)** Phylogenetic relationship of NUDX proteins. Numbers on branches indicate percentage of replicate trees in which the associated taxa clustered together in the bootstrap test (1000 replicates). GenBank accessions of NUDX proteins were provided in Supplementary Table S2. **(B)** Subcellular localization analysis of PlNUDX. Bright light, bright field image; green, GFP fluorescence detected in the green channel; magenta, chlorophyll autofluorescence; merged, merged green and magenta channel images. Bars = 25 μm. **(C)**  *In vitro* enzyme activity analysis of PlNUDX. **(D)** Transient transformation of *PlNUDX* into *N. benthamiana* leaves. Volatiles were analyzed by HS-SPME-GC–MS. **(E)** Ion chromatograms showing the VOCs detected by GC–MS in *PlTPS9*-silenced plants. **(F)** Ion chromatograms showing the VOCs detected by GC–MS in *NUDX*-silenced plants. **(G)** Relative quantification of VOCs emitted from *PlTPS9*-silenced *P. lactiflora* flowers. **(H)** Relative quantification of VOCs emitted from *NUDX*-silenced *P. lactiflora* flowers. Data in G and H are means ± SEM, *n* = 8. Detailed data in G and H can be found in Supplementary Table S7. **(I)**  *PlTPS9* transcript levels in *P. lactiflora* flowers detected by RT-qPCR quantification. **(J)**  *NUDX* transcript levels in *P. lactiflora* flowers detected by RT-qPCR quantification. Data in I and J are means ± SEM from at least three biological replicates. The volatiles were analyzed by HS-SPME-GC–MS. Compound identification was performed by comparing spectra with those in the NIST-2017 mass spectral library.

Cytosolic NUDXs have been shown to contribute directly to the production of monoterpene alcohols, such as geraniol in roses [8, 19] and geraniol, citronellol in rose-scented geranium [18]. Overexpression of RrNUDX1 in *Petunia* established that *NUDX* genes enhance volatile biosynthesis and increase geraniol and related compounds [69]. In a similar manner, monoterpenoid biosynthesis likely occurs via a cytosolic pathway in *P. lactiflora*, where specific enzymes hydrolyze GPP, indicating a compartmentalized mechanism distinct from the plastid-localized TPS route. Accordingly, we characterized NUDX activity *in vitro* and *in vivo*. In brief, PlNUDX was identified by aligning putative genes with well-known *NUDX* genes from other plant species (Supplementary Fig. S12). Subsequently, phylogenetic analysis showed PlNUDX protein clustered closely with the NUDX identified from *Pelargonium graveolens* (Fig. 6A), suggesting a possible functional similarity. Subcellular localization analysis revealed that PlNUDX was localized predominantly in the cytosol, which was consistent with the prediction generated by the TargetP-2.0 (Fig. 6B; Supplementary Table S6).

To characterize the enzymatic activity of PlNUDX, we heterologously expressed the gene in a recombinant system and purified soluble PlNUDX protein. The purified enzyme was incubated with the isoprenoid diphosphate substrates GPP and NPP, and reaction products were analyzed via ultraperformance liquid chromatography tandem mass spectrometry (UPLC-MS/MS). Compared with control reactions lacking PlNUDX, the recombinant protein catalyzed the hydrolysis of GPP to GP and the conversion of NPP to NP (Fig. 6C). To validate the *in vivo* function of PlNUDX, we coexpressed *PlNUDX* with either GPP-synthesizing *PlGPPS* or NPP-producing *SlNPPS* in *N. benthamiana* leaves. This coexpression resulted in the obvious biosynthesis of geraniol and nerol, respectively, confirming PlNUDX’s ability to generate monoterpene alcohols *in planta* (Fig. 6D; Supplementary Table S5). Collectively, these results demonstrate that PlNUDX provides an alternative route for monoterpene alcohol biosynthesis, independent of canonical TPSs. Its ability to accept both GPP and NPP as substrates underscores its substrate promiscuity—a trait that may confer adaptive advantages under fluctuating metabolic flux conditions.

Of note, PlTPS9 also catalyzes the formation of geraniol (Figs 3 and 4). To verify the functional divergence or redundance of *PlNUDX* and *PlTPS9* genes in peony, two gene fragments with ~400–600 bp were selected. Following RNAi-mediated silencing of *PlTPS9* and *PlNUDX* genes in peony petals, volatile analysis revealed distinct alterations in terpene production compared to the control. In petals infiltrated with *PlTPS9*-*RNAi* constructs, the levels of citronellol and geraniol were markedly reduced, consistent with effective gene silencing (Fig. 6E and G; Supplementary Table S7). Similarly, RNA interference of *NUDX* also led to a significant decrease in citronellol and geraniol production when compared to the control group (Fig. 6F and H; Supplementary Table S7). Consistent with the reduction of geraniol and its derivatives, the expression of *PlTPS9* and *PlNUDX* were downregulated in the RNAi petals (Fig. 6I and J). On the contrary, the emission of β-caryophyllene was not changed. These results suggest that PlTPS9 and PlNUDX play overlapping roles in regulating monoterpene biosynthesis in distinct subcellular compartments, particularly citronellol and geraniol, without significantly affecting sesquiterpene production.

### Prenyltransferases supply precursor substrates for key floral volatile biosynthesis in *P. lactiflora* ‘Zifengyu’

**Figure 7:**
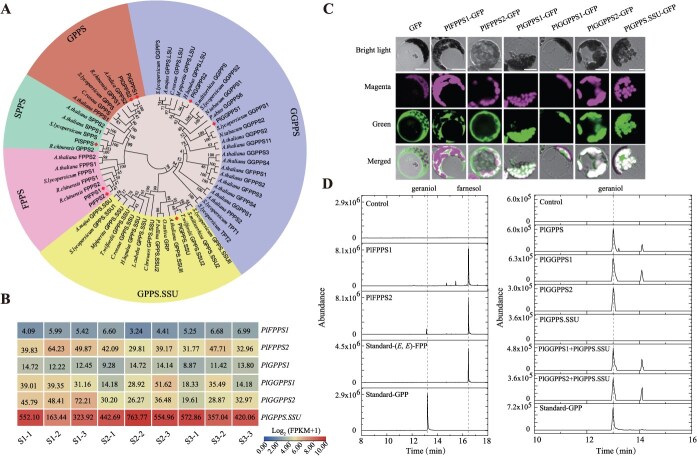
Exploration of the substrate sources that control the synthesis of major volatile terpenoids in the *P. lactiflora* ‘Zifengyu’. **(A)** Phylogenetic analysis of identified PTs proteins in peony and other plants. Numbers on branches indicate percentage of replicate trees in which the associated taxa clustered together in the bootstrap test (1000 replicates). TPS proteins from *P. lactiflora* ‘Zifengyu’ were indicated by solid rhombus pattern. GenBank accessions of other TPS proteins were provided in Supplementary Table S2. **(B)** Relative expression levels of *PTs* genes at different stages of peony flower opening. The data from FPKM values of RNA-Seq. **(C)** Subcellular localization of peony PTs proteins observed in *Arabidopsis* protoplasts. Bright light, bright field image; green, GFP fluorescence detected in the green channel; magenta, chlorophyll autofluorescence; merged, merged green and magenta channel images. Bars = 25 μm. **(D)**  *In vitro* enzyme activity assay of peony PTs proteins. GPP and (*E, E*)-FPP standards were hydrolyzed to their corresponding alcohols, geraniol and farnesol, respectively. The volatiles were analyzed by HS-SPME-GC–MS. Compound identification was performed by comparing spectra with those in the NIST-2017 mass spectral library.

The biosynthesis of volatile isoprenoids is mediated by PTs, which condense IPP and DMAPP into essential intermediates such as GPP, FPP, and GGPP. Through transcriptome analysis and sequence alignment, eight *PT* genes were identified in peony. Phylogenetic analysis revealed high sequence similarity between these PTs and characterized homologs from other plant species, suggesting conserved function (Fig. 7A). Among the identified genes, *PlGPPS.SSU* exhibited consistently high expression levels across all floral developmental stages, implicating it in sustained monoterpene production. Meanwhile, *PlFPPS2* also showed strong and widespread expression across various tissues and developmental stages in contrast to *PlFPPS1* (Fig. 7B). Subcellular localization studies using GFP fusion constructs in *Arabidopsis* protoplasts showed that enzymes associated with the MEP pathway (PlGPPS, PlGGPPS, and PlGPPS.SSU) localized to plastids, whereas MVA pathway-related enzymes (PlFPPS1 and PlFPPS2) localized in the cytosol (Fig. 7C), which is largely consistent with TargetP-2.0 localization predictions, with PlGPPS as the sole deviation (Supplementary Table S6). These findings support a compartmentalized model of terpene biosynthesis, with monoterpene precursors produced plastidially and sesquiterpene precursors synthesized cytosolically.

Functional enzymatic assays using recombinant proteins expressed in *Escherichia coli* further confirmed the substrate specificities of these PTs. PlFPPS1 catalyzed the synthesis of FPP exclusively, while PlFPPS2 could synthesize both FPP and trace amounts of GPP. This dual activity of PlFPPS2 provides a likely cytosolic source of GPP, which may explain the presence of geraniol-derived volatiles in cytosol-derived monoterpene pathways [8]. In contrast, both PlGPPS and PlGGPPS efficiently catalyzed GPP formation from IPP and DMAPP. However, PlGPPS.SSU failed to enhance GPP synthesis (Fig. 7D).

## Discussion

### The genetic blueprint of herbaceous peony floral volatile terpenes has been well decoded

According to their origination pathway, floral VOCs are usually divided into three categories, including terpenoids, benzenoids, and phenylpropanoids, and fatty acid derivatives. Till now, hundreds of herbaceous peony cultivars have been sent to floral VOC analysis, volatile terpenes and their ester derivatives are identified as prevailing volatiles, such as β-caryophyllene, geraniol, nerol, citronellol, neryl acetate, citronellyl acetate, 1, 8-cineole, and linalool [58, 60, 62, 70, 71]. Consistently, β-caryophyllene, geraniol, citronellol, and 1, 8-cineole are most abundantly emitted from flowers of *P. lactiflora* ‘Zifengyu’, and the distribution of volatile terpenoids across floral organs and developmental stages reveals a tightly regulated spatial and temporal biosynthesis pattern. For instance, β-caryophyllene accumulates predominantly in sepals, ovaries, and early flower developmental stages suggesting a defensive function against herbivores and pathogens [72]. In contrast, geraniol, citronellol, 1, 8-cineole, and (*Z*)-β-ocimene are highly emitted from stamens and petals as well as flowers at late developmental stages, aligning with their potential roles in pollinator attraction [73]. This shift from sesquiterpenoid dominance in early stages to monoterpenoid prevalence during anthesis reflects a metabolic reprogramming to support reproductive success. Similar scenarios are also observed in *P. lactiflora* ‘Wu Hua Long Yu’, highlighting the conserved roles of floral volatiles in herbaceous peonies [66].

TPS acts as the metabolic gatekeeper in the biosynthesis of the diverse terpenoids, which has been widely investigated [14, 23], seven subfamilies have been set up based on their phylogenetic relationships in plant lineages [74]. Although members of TPSa and TPSb subfamilies are generally ascribed to be monoterpene and sesquiterpene synthases, respectively, and proteins from TPSg subfamily catalyze the production of both monoterpene and sesquiterpene simultaneously, increasing evidence indicates the substrate promiscuity of the TPS proteins regardless of their subfamilies, which can utilize multiple prenyl diphosphates to generate a plethora of products [8, 10, 75–78]. Consistently, six of the nine identified PlTPS proteins were identified to use four configurations of prenyl diphosphates to generate multiple products (Fig. 3). The widely distributed substrate promiscuity of TPS proteins implies that the catalytic feature of angiosperm-specific TPS proteins might evolve early before the diversification of TPS-a, b, and g subfamilies, whereas their origin, evolution, and functional divergence remain obscure. Previously, TPS proteins with geraniol, linalool, α-pinene, β-ocimene, and β-caryophyllene as major or single product have been identified [63, 65, 66, 79], allelic variants of those TPS proteins were further confirmed in this study, which have different product profiles because of amino acid residues polymorphisms (Supplementary Figs S1–S9). In addition, PlTPS5 was found to control the biosynthesis of 1, 8-cineole, which was confirmed in this study. In summary, TPS proteins accounting for the already detected major volatile terpenes in herbaceous peonies have been successfully isolated, while more efforts still need to be made when more herbaceous peony resources are employed and the annotation of their genomes are completed. Besides PlTPS9, an alternative routine to synthesize geraniol depending on PlNUDX was also verified herein (Fig. 6), and the conversion of geraniol to citronellol has also been unraveled recently [8, 80, 81]. However, the acylation modification of geraniol and citronellol still requires further resolved. Moreover, eight PTs were also functionally characterized in this study to shed light on the substrate supply of volatile terpenes, results suggest that monoterpene precursors (GPP) are primarily synthesized in plastids and less in cytosol, while sesquiterpene precursors (FPP) arise from the cytosol (Fig. 7). Therefore, the genetic blueprint of herbaceous peony floral volatile terpenes has been well decoded from substrate supply to biosynthesis and derivatization processes.

### Amino acids residues participate in determining the TPS4 and TPS5 catalytic specificity

Generally, the enzymatic reactions of TPSs are considered exceptionally complex in nature, mechanisms underlying involve ionization, proton loss, and water capture, resulting in the synthesis of various cyclization products that drive the chemodiversity of volatile terpenes in plants [82, 83]. Alanine-scanning mutagenesis coupled with enzyme fidelity analysis of (*4S*)-limonene revealed that the active site pocket is enclosed mostly by aromatic amino acids residues. In other reported studies, the occurrence of multiple aromatic residues adjacent to the active site pocket of TPSs is critical for the stability of carbocation radical species [84, 85]. Superimposition of TPS4 with TEAS template revealed the key residues at position W283, D311, and R453 residues, while Asn338 in SfCinS1 known to capture water molecules during catalysis align with TPS5-Asn323 (Fig. 5; Kampranis et al., 2007). In order to study the roles of the active site amino acids in defining the respective product specificities of TPS4 and TPS5, we created mutants containing suspected single amino acid. Consequently, site-directed mutagenesis of PlTPS4 and PlTPS5 identified critical residues influencing substrate selectivity and product formation. For example, W283 and R453 in PlTPS4 and Asn-323 in PlTPS5 were shown to be key in determining monoterpene vs sesquiterpene product specificity, respectively. Similarly, several studies have shown that changing a single amino acid in the active pocket influences the products of sesqui-TPSs such as SbTPS1 and SbTPS2 in *Sorghum bicolor* [86]*,* maize TPS4, and TPS10 [87], monoterpene synthases in dipterocarpaceae [88]. These insights could be exploited for rational enzyme engineering to alter scent profiles in ornamental plants. Generally speaking, although the catalytic efficient of mutant may be altered compared to wild-type enzymes, these findings suggest that plasticity residues affect the enzyme activity and product selectivity. While some plasticity residues may not directly interact with the substrate, further investigation into their impact on enzyme function will provide valuable insights into the biochemical mechanisms behind terpene chemodiversity and could inform strategies for enzyme bioengineering toward floriculture improvement.

### PlNUDX is likely as an alternative geraniol and citronellol biosynthetic pathway in *P. lactiflora*

Previous studies demonstrate that roses and pelargonium utilize a TPS-independent NUDX pathway for geraniol production [18–20, 38]. Notably, citronellol originates from geraniol, catalyzed by a series of enzymes from diverse families in cytosol compartment [8, 11, 89–91]. In many rose resources, *TPS* genes have become lowly expressed or pseudogenized in floral tissues, likely due to reduced selection pressure as the NUDX pathway became the primary monoterpene biosynthesis route [7, 8, 38]. Therefore, we hypothesized that in *P. lactiflora,* monoterpenoid biosynthesis will likely occur via mechanisms independent from the plastid-localized terpene synthase route. Importantly, the biosynthesis of geraniol through TPS-independent pathway remains unclear, warranting investigation due to their vital roles in pollination, defense, and commercial applications. In this study, PlNUDX functions as a Nudix-type phosphatase that directly contributes to floral geraniol biosynthesis in *P. lactiflora*. Enzymatic assays combining PlNUDX with GPP and NPP in the presence of alkaline phosphatase led to the production of geraniol and nerol confirming its ability to mediate hydrolytic release of volatile alcohol precursors. As previously described in other plant species, these findings position PlNUDX as a versatile enzyme that catalyzes a noncanonical, TPS-independent route for terpene production in peony [20, 69]. In addition, the involvement of NUDX in monoterpene biosynthesis likely operates within a complex, flexible enzymatic network, as the hydrolytic pathway mediated by NUDX may function in concert with auxiliary enzymes such as geraniol dehydrogenase (GeDH), geranial reductase (GER), citronellal reductase (CAR) and 12-oxophytodienoate reductase (OPR), collectively contributing to the biosynthesis and structural modification of monoterpene derivatives, and all the enzymes are localized in cotysol compartment [8], supporting the idea that cytosolic GPP from bifunctional G/FPPS1 is crucial in species like rose [7] and possibly peony (Cytosolic PlFPPS2 generated minor quantity of GPP, Fig. 7). However, nerol and farnesol are not emitted from *P. lactiflora* ‘Zifengyu’ flowers, which seems to be contradictory with the versatile roles of NUDX [18, 20, 83]. The absence of nerol and farnesol emission in peony flowers likely stems from a combination of substrate limitation, metabolic flux competition, downstream modification, and uncharacterized regulatory mechanisms [18, 83, 92, 93]. Firstly, peonies likely lack functional neryl diphosphate synthase (NPPS), eliminating the NPP substrate required for nerol biosynthesis. Secondly, even if low NPP or FPP levels exist, TPS and essential pathways (e.g. sterol synthesis) may sequester these substrates for signature volatiles and housekeeping functions. Thirdly, any transient nerol/farnesol may be rapidly converted to nonvolatile glycosides or oxidized derivatives. Forthly, uncharacterized mechanisms—including post-translational modification of PlNUDX, subcellular substrate–enzyme separation, or feedback inhibition—may further restrict alcohol production, collectively shaping peony’s unique scent profile.

Overall, all these findings suggest an adaptive shift toward NUDX-mediated hydrolysis as an alternative route for producing key floral volatiles like geraniol, citronellol, and nerol. In summary, future comparative studies evaluating *NUDX* expression, catalytic efficiency, and evolutionary conservation across monoterpene-rich taxa such as rose, pelargonium, and tomato could reveal the extent to which this alternative terpene biosynthesis strategy is conserved or lineage-specific [17].

## Conclusion

Despite the ornamental and economic value of *P. lactiflora*, the molecular underpinnings of its complex floral scent, especially the biosynthesis of key terpenoid volatiles, remain poorly understood. A comprehensive understanding of TPS and TPS-independent NUDX function, precursor biosynthesis and subcellular metabolic coordination is essential for dissecting and manipulating floral volatile pathways. In this study, we identified and functionally characterized 12 *TPS* genes, out of which nine (PlTPS1–PlTPS9) were catalytically active in both *in vitro* and *in planta* systems. These TPSs displayed diverse product profiles and substrate preferences, with key compounds such as β-caryophyllene, 1, 8-cineole, linalool, and geraniol emerging as dominant floral volatiles. Notably, PlTPS4 and PlTPS5 were confirmed as signature synthases for β-caryophyllene and 1, 8-cineole biosynthesis, respectively, with mutational analyses revealing critical amino acid residues essential for their enzymatic activity. In addition, the identification of PlNUDX, a cytosolic Nudix hydrolase capable of hydrolyzing GPP and NPP to produce geraniol and nerol, presents an alternate monoterpenoid biosynthetic pathway independent of TPSs, adding further biochemical diversity to peony scent production. Functional validation of recombinant PT enzymes supported their role in precursor generation, with PlFPPS2 exhibiting dual catalytic capacity for FPP and trace GPP, potentially linking cytosolic terpenoid biosynthesis with geraniol formation via noncanonical routes. Although transient expression and *in vitro* assays are informative, stable genetic transformation and *in situ* metabolomic tracking in *P. lactiflora* would provide stronger functional validation. Moreover, compound identification in this study relied on spectral matching against the NIST-2017 mass spectral library—a routine approach for VOC analysis—yet has inherent limitations. Specifically, it lacks certainty in identifying rare compounds (e.g. acoradiene, ledene) and distinguishing enantiomers. Future research should incorporate authentic standards or structural characterization techniques (e.g. nuclear magnetic resonance, NMR) to enhance the accuracy and reliability of compound identification.

In summary, our study reveals a highly coordinated, compartmentalized, and genetically diverse terpene biosynthesis network in *P. lactiflora* ‘Zifengyu’, driven by distinct TPS, PT, and NUDX enzymes. These insights not only deepen our understanding of floral scent biology but also pave the way for future innovation in scent engineering and ornamental plant improvement.

## Materials and methods

### Experimental materials

The *P. lactiflora* ‘Zifengyu’, plants used in this study were cultivated in the Peony Garden of Northeast Normal University (Changchun, Jilin province, China; GPS coordinates of 43°53′28.5432″N, 125°19′52.8420″E). The *P. lactiflora* ‘Zifengyu’ flowers at different developmental stages (budding, half-open, and fully open) were selected for volatile analyses with the earlier well-established headspace solid-phase microextraction (HS-SPME) method, followed by GC–MS analysis as described below [16]. Additionally, flowers at the first day of anthesis were dissected into petals, stamens, sepals, and carpels, which were further analyzed via HS-SPME-GC–MS. All samples were weighted for subsequent quantitative analysis before being frozen in liquid nitrogen, and stored at −80°C until use.

*Arabidopsis thaliana* and *N. benthamiana* plants were grown in a greenhouse under a 16 h light and 8 h dark photoperiod at 22°C. Four- to 6-week-old *Arabidopsis* and *N. benthamiana* plants were subjected to protoplast isolation and bacterial infiltration, respectively.

### HS-SPME-GC–MS analysis of volatile compounds

Samples were sealed in an odor-free plastic device or glass vials. Volatiles captured by a 100-mm divinylbenzene/carboxen/polydimethylsiloxane (DVB/CAR/PDMS) (Sigma Aldrich, USA) fused silica fiber (Sigma-Aldrich, USA) used in HS-SPME were directly analyzed by GC–MS following thermal desorption at 250°C for 4 min to ensure complete release. Subsequently, the desorbed volatiles were carried by high-purity nitrogen gas (>99.99%) into GC column oven. The oven temperature program was set as follows: initial temperature 60°C (held for 3 min), then ramped to 100°C at 5°C/min (held for 1 min) and finally increased to 250°C at 10°C/min (held for 10 min). Mass spectra were recorded in electron impact (EI) mode and compound identification was performed by comparing spectra with those in the NIST-2017 mass spectral library.

### Nucleic acid preparation, gene cloning, and expression analysis

Total RNA was extracted using the OminiPlant RNA Kit (CWBIO, Jiangsu, China) according to the manufacturer’s instructions. RNA quality and concentration were evaluated with a Nano-500 microspectrophotometer (Allsheng Instruments, Hangzhou, China). First-strand cDNA was synthesized from the RNA sample using the UELris II RT-PCR System for First-Strand cDNA Synthesis (with dsDNase) Kit (US Everbright® Inc., Suzhou, China) and the resulting cDNA was used for gene cloning and expression analysis.

Genes encoding TPS, NUDX, and PT enzymes from other plant species were used as queries to screen a previously established transcriptome database (Supplementary Table S2) for putative homologous genes via manual BLAST searches integrated in TBtools-II [67]. Subsequently, cDNA sequences were subjected to manual NCBI-BLASTX search, and the best hits were taken as putative genes (Supplementary Table S8). Subsequently, specific primers (Supplementary Table S3) were designed for full-length gene cloning in routine PCR and gene expression analysis in reverse transcription quantitative PCR (RT-qPCR). Gene expression levels were either assessed by reads per kb per million reads (RPKM) or analyzed by RT-qPCR using a CFX96™ Real-Time PCR Detection System (BIO-RAD, Hercules, CA, USA). For RT-qPCR, SYBR Green-based PCR assays were performed in a total volume of 10 μl reaction mixture containing 5 μl of TB Green® Premix Ex Taq (TaKaRa, Kusatsu, Japan), 0.5 μM each primer and 1 μl of cDNA template. *PlACTIN* and *PlUBI* were used as internal reference genes [88, 94]. Detailed amplification conditions were as previously reported [95].

### Sequence alignment and phylogenetic analysis

Multiple sequence alignments were conducted using the Clustal Omega algorithm (https://www.ebi.ac.uk/Tools/msa/clustalo/) with default parameters. Phylogenetic tree was constructed using the plugin One Step Build an ML Tree integrated in TBtools-II with robustness evaluated via bootstrap resampling analysis (1000 replicates).

### Subcellular localization analysis

For the experimental procedures of subcellular localization: Rossette leaves from 4-week-old *Arabidopsis* plants were used for protoplast isolation following established protocols [95, 96]. Briefly, the leaves were cut into strips and digested with Cellulase R10 and Macerozyme R10 (both from Yakult Pharmaceutical Ind. Co., Ltd., Japan) to release protoplasts. The protoplast suspension was then filtered through Miracloth (EMD Millipore Corp., Billerica, MA, USA) and washed by centrifugation to remove residual enzyme solution. Purified protoplasts were resuspended in MMG solution [96] and prepared for subsequent transformation.

The coding sequences (CDSs) of target genes were seamlessly cloned into *Nde*I and *Cla*I digested *35S: FhMYBPA1-GFP* vector [97] using the pEASY®-Basic Seamless Cloning and Assembly Kit (TransGen Biotech, Beijing, China), generating recombinant plasmids *35S*: *PlTPSs/PlNUDX/PlPTs-GFP*. The plasmids were further purified using the endotoxin-free GoldHi EndoFree Plasmid Maxi Kit (CWBIO, Beijing, China) to remove endotoxins. Protoplast transformation was performed via PEG3350-mediated transfection, followed by incubation in the dark for 22 h. Transfected protoplasts were observed under a laser confocal microscope (Olympus, Japan) with excitation and emission wavelengths set at 488 and 546 nm, respectively.

For the online prediction of subcellular localization: subcellular localization of proteins was predicted via the TargetP-2.0 program. The amino acid sequences were standardized to the FASTA format for submission, with the Organism group set to Plant and Output format chosen as Long output—a setting that guarantees one graphical plot and one summary for each protein sequence.

### *In vitro* enzymatic assay

The CDSs of target genes were seamlessly cloned into *BamH*I and *EcoR*I digested pET32a vector and subsequently transformed into *E. coli* strain BL21 (DE3) competent cells (Coolaber, Beijing, China). Recombinant proteins were induced with isopropyl-β-D-thiogalactopyranoside (IPTG) at 16°C for 18 h and purified using Ni-TED Sefinose™ column (Sangon Biotech, Shanghai, China) following published protocols [10, 95].

*In vitro* recombinant TPS activity assays were performed according to published methods [8, 10]. Briefly, a 300 μl reaction mixture containing 25 mM HEPES (pH 7.4), 10% (v/v) glycerol, 7.5 mM MgCl_2_, 3.3 mM KCl, 0.6 mM MnCl_2_, 5 mM DTT, and 2 mM GPP/NPP/(*E, E*)-FPP/(*Z, Z*)-FPP was incubated with 100 μg of purified recombinant proteins in a 30°C water bath for 2 h. Volatiles were captured using SPME fibers followed by GC–MS analysis. The relative content of enzyme products were calculated relative to standard β-caryophyllene [10], and the unit was ng.

PT activity assays were performed as described in our published paper [8]. The reaction mixture contained 100 μg of recombinant PT proteins, 25 mM HEPES (pH 7.4), 10% (v/v) glycerol, 10 mM MgCl_2_, 10 mM KCl, 1.25 mM DTT, 1.25 mM sodium ascorbate, and 100 μM IPP, DMAPP. The mixture was incubated in a 30°C water bath for 2 h, then digested with calf intestinal alkaline phosphatase (CIP) at 37°C for 3 h, while simultaneously adsorbing reaction products using SPME fibers. The fibers were desorbed in the GC–MS system to detect the enzymatic products.

NUDX activity assays were performed following published papers [8]. The reaction mixture contained 100 μg of NUDX proteins, 2 mM GPP/NPP, 5 mM MgCl_2_, 1 mM MnCl_2_, 14 mM β-mercaptoethanol, 50 mM HEPES (pH 8.0), and 10% glycerol. The mixture was incubated in a 30°C water bath for 2 h. To denature the protein, 20 μl of precooled acetonitrile was added, followed by centrifugation at 3000 g for 5 min. The aqueous phase was then transferred for analysis using ultrahigh-performance liquid chromatography electrospray ionization tandem mass spectrometry (UPLC-ESI-MS) system equipped with an Agilent Eclipse Plus RRHD C18 column (2.1 × 50 mm, 1.9 μm particle size). A full-scan acquisition mode was employed with an 8-min gradient elution protocol. The mobile phase consisted of solvent A (5 mM ammonium bicarbonate in water) and solvent B (acetonitrile). The gradient program was as follows: initial conditions (95% A, 5% B) were held, then linearly decreased to 30% A (70% B) over 5 min, followed by a rapid increase to 95% B at 6 min, held for 30 s, and returned to initial conditions within 1.5 min for reequilibration. Total run time per sample was ~8 min. The injection volume was fixed at 5 μl, and each sample was analyzed in triplicate to ensure reproducibility.

### Structure model construction and molecular docking

The structure models of target proteins were predicted by AlphaFold (https://alphafold.ebi.ac.uk; [98]) while GPP (ID: 445995) and (*E, E*)-FPP (ID: 445713) were obtained from Pubchem databases (https://pubchem.ncbi.nlm.nih.gov/). The terpene synthase protein and ligands (GPP and FPP) were prepared using AutoDock Vina protocols and docking results were analyzed with ChimeraX to predict ligand–protein interactions. *Salvia fruticosa* 1, 8-cineole synthase (PDB ID: 2J5C) [99], and *N. tabacum* epi-aristolochene synthase TEAS (PDB ID: 3 M00) [100] with reported crystal structures were downloaded from the PDB database (https://www.rcsb.org/). Subsequently, docked structures were superimposed with resolved structure as a template. Visualization of the docking models and corresponding graphical outcomes was implemented by UCSF ChimeraX (https://www.cgl.ucsf.edu/chimerax/). To localize the pockets and their adjacent amino acids residues, atoms within 3 Å of the docked ligand were considered. To verify the function of predicted key amino acids, the PCR-based mutagenesis was executed by the Fast Mutagenesis System (TransGen Biotech Ltd., Beijing, PRC) following the manufacturer’s instruction. Constructs harboring the mutations were verified by sequencing before expression. Overall, the mutated proteins were expressed and subsequently purified aforementioned and subject to further enzymatic assays [10, 16].

### *In vivo* gene overexpression in *N. benthamiana* or silence in peony

The functional characterization of target genes in *N. benthamiana* leaves was conducted according to the method described earlier [10, 16]. Briefly, the full-length CDSs of target genes were subcloned into *Nru*I and *Xho*I digested pEAQ-HT [101] and subsequently transformed into *A. tumefaciens* GV3101 competent cells (Coolaber, Beijing, China). Positive clones were screened and inoculated into LB liquid medium supplemented with appropriate antibiotic. When the bacterial culture reached an optical density at 600 nm (OD_600_) of 0.6–0.8, the culture was centrifuged and washed several times to remove antibotic. The bacterial pellet was finally resuspended in infiltration buffer containing 1 mM MES, 1 mM MgCl_2_, and 15 ng/ml acetosyringone (AS). The suspension was diluted to an OD_600_ of 0.8, incubated at room temperature for 2 h, and then infiltrated into abaxial surface of *N. benthamiana* leaves using a sterile syringe. After 4 days of infiltration, ~1 g of infiltrated leaves was collected and transferred to HS-SPME-GC–MS.

For RNA interference (RNAi)-mediated silencing of *PlNUDX* and *PlTPS9*, sense and antisense CDS fragments of the target genes were subcloned into the RNAi vector pFGC5941M [102] to allow the formation of hairpin RNA (hpRNA) structures. Subsequently, the recombinant vectors were transformed into *A. tumefaciens* strain GV3101 competent cells. Positive clones were screened and prepared following the previously described method. The final bacterial suspension was adjusted to an OD_600_ of 0.8 and infiltrated into *P. lactiflora* petals. For each peony flower, one-half was infiltrated with *A. tumefaciens* harboring the empty pFGC5941M vector as the control group, and the other half with *A. tumefaciens* harboring the target gene RNAi vector as the experimental group. Three days later, the infiltrated petal samples were collected and subjected to subsequent volatile compound analysis and gene expression profiling.

For the control group normalization, among the eight control replicates (each derived from one-half of a longitudinally split flower), the peak area of the target volatile from one representative control sample was set as the 100% reference. The peak areas of the remaining seven control samples were then calculated as relative percentages against this reference value. For each treatment sample (the paired half of the same split flower), its peak area was normalized relative to the peak area of its corresponding control sample (from the same flower). Consequently, the mean values and standard deviations (represented as error bars) were computed across all eight replicate samples (both control and treatment groups) to reflect the experimental variability.

## Consent for Participation and Publication

All authors approve the manuscript and consent to publication of the work.

## Supplementary Material

Web_Material_uhag091

## Data Availability

All metabolite and transcriptome data, which were generated or analyzed during this study, were included in this published article and its supplementary information files (Tables S1 & S4–S8). Moreover, all other data are available from the corresponding author upon reasonable request.
