# Supplementary material for: Genetic blueprint of herbaceous peony floral scent: evidence from terpene synthase, Nudix hydrolase, and prenyltransferase
Source: Hortic Res. 2026 Mar 9;13(7):uhag091. doi: 10.1093/hr/uhag091 (PMC13278846; doi:10.1093/hr/uhag091)
Supplement: Web_Material_uhag091 [file web_material_uhag091.zip › Supplementary20260225.docx]

**Genetic Blueprint of Herbaceous Peony Floral Scent: Evidence from Terpene Synthase, Nudix Hydrolase and Prenyltransferase**

Tingting Bao^a,bϯ^, Kimani Shadrack^c,ϯ^, Xiaotong Shan^b^, Hongjie Li^b^, Luhong Leng^b^, Yueqing Li^b*^, Zhiqiang Wu^a*^, Xiang Gao^b*^

^a^ State Key Laboratory of Tropical Crop Breeding, Shenzhen Branch, Guangdong Laboratory of Lingnan Modern Agriculture, Key Laboratory of Synthetic Biology, Ministry of Agriculture and Rural Affairs, Agricultural Genomics Institute at Shenzhen, Chinese Academy of Agricultural Sciences, Shenzhen, 518120, China

^b^ Key Laboratory of Molecular Epigenetics of MOE and Institute of Genetics & Cytology, Northeast Normal University, Changchun, 130024, China.

^c^ Karatina University, School of Pure and Applied Sciences, 1957-10101, Karatina, Kenya.

**Running Title:** **Biogenesis of floral volatile terpene in herbaceous peony**

* Corresponding authors: To whom correspondence should be addressed.

Email address: gaoxiang424@163.com; [wuzhiqiang@caas.cn;](mailto:wuzhiqiang@caas.cn;) liyq339@126.com.

^ϯ^The authors contributed equally to this work.


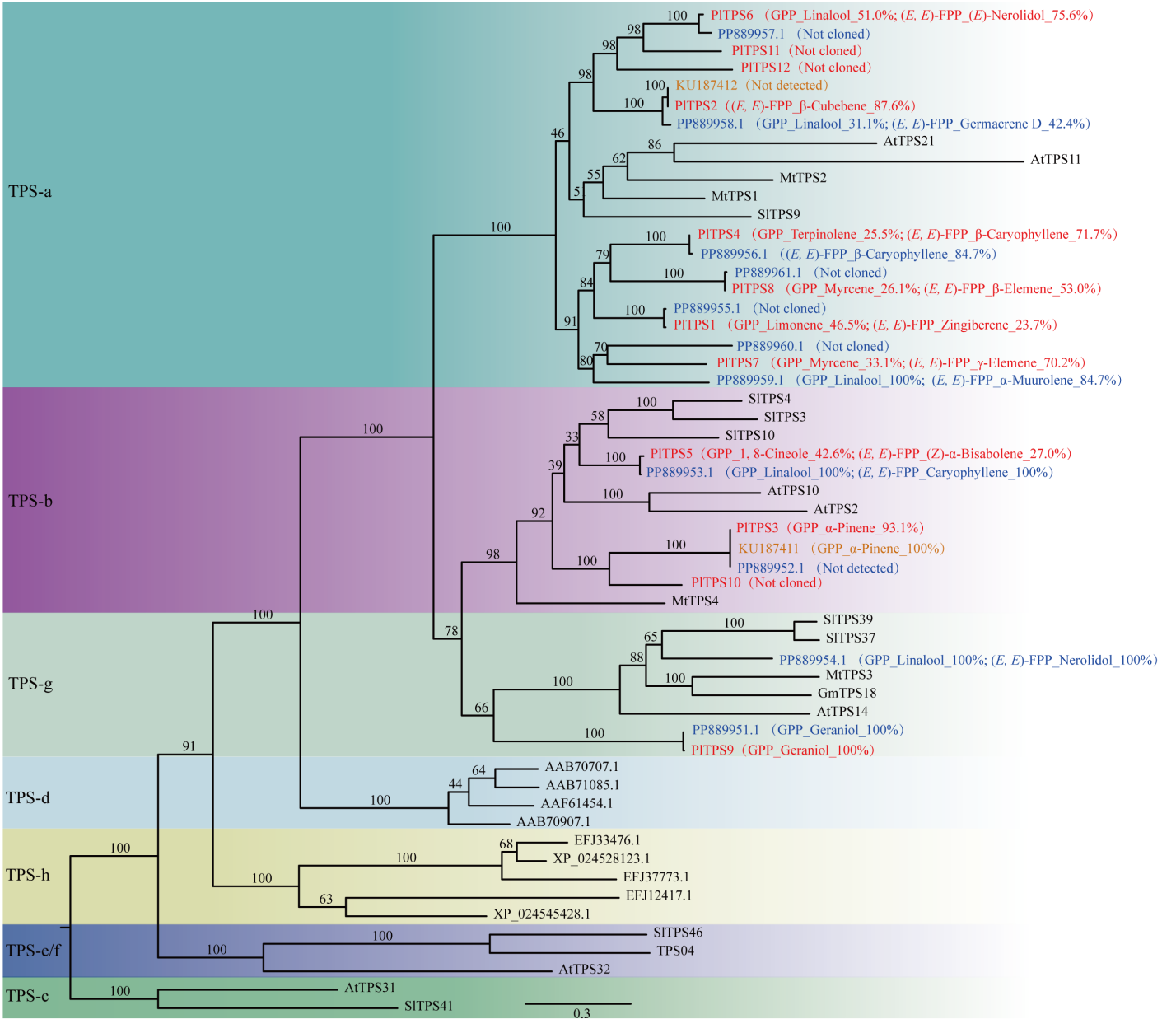


**Supplementary Figure S1.** Maximum likelihood tree (MLtree) of TPS proteins and functional comparison with other reported TPSs. Numbers on branches indicate percentage of replicate trees in which the associated taxa clustered together in the bootstrap test (1,000 replicates). The tree was drawn to scale, with branch lengths in the same units as those of the evolutionary distances used to infer the phylogenetic tree. The evolutionary distances were computed in the units of the number of amino acid substitutions per site, as shown by the scale below the tree. TPS proteins from *P. lactiflora* ‘Zifengyu’ were indicated by red front, and TPS proteins from *P. lactiflora* ‘Wu Hua Long Yu’ (Zhao et al., 2025b) were indicated by blue front, KU187411 and KU187412 from the reference published in 2016 (Ma et al., 2016) were indicated by yellow front. GenBank accessions of other TPS proteins were provided in Supplementary Table S2.


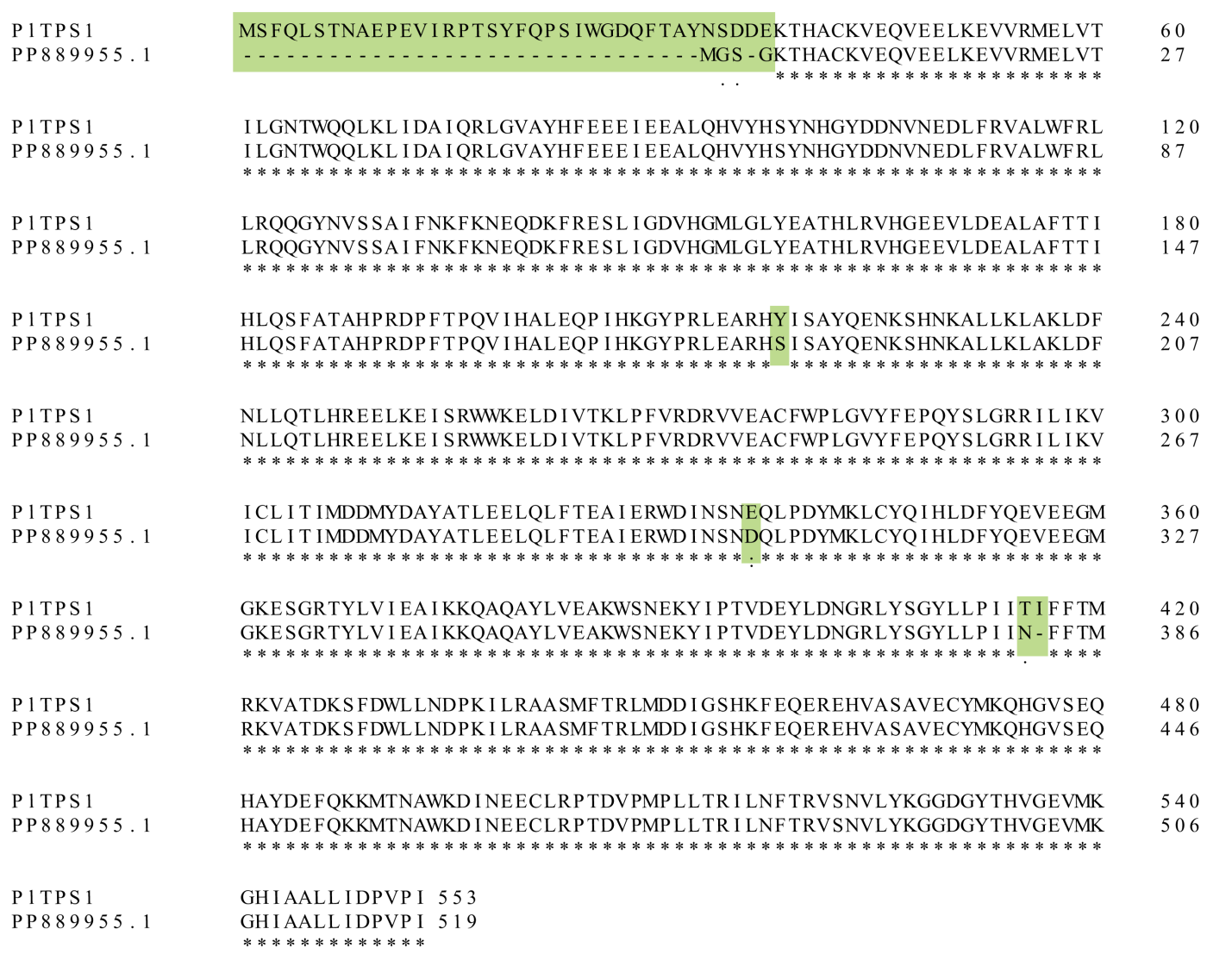


**Supplementary Figure S2.** Sequence alignment of TPS1 from *P. lactiflora* ‘Zifengyu’ and PP889955.1 from *P. lactiflora* ‘Wu Hua Long Yu’. The residue polymorphism were highlighted with green backgrounds. Numbers represent positions of specific residues. *, identical amino acids, : or ·, similar amino acids.


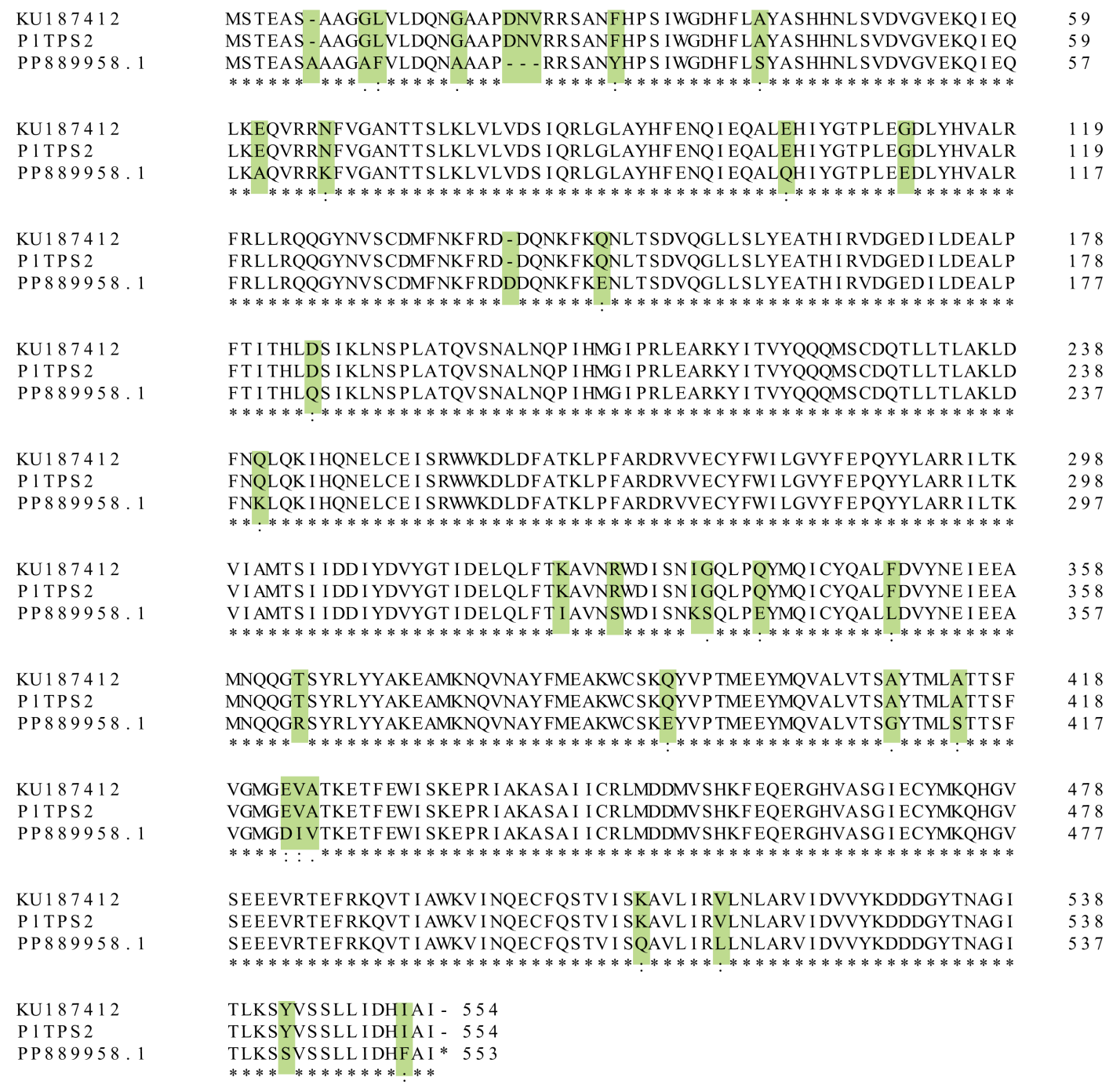


**Supplementary Figure S3.** Sequence alignment of KU187412, TPS2 from *P. lactiflora* ‘Zifengyu’ and PP889958.1 from *P. lactiflora* ‘Wu Hua Long Yu’. The residue polymorphism were highlighted with green backgrounds. Numbers represent positions of specific residues. *, identical amino acids, : or ·, similar amino acids.


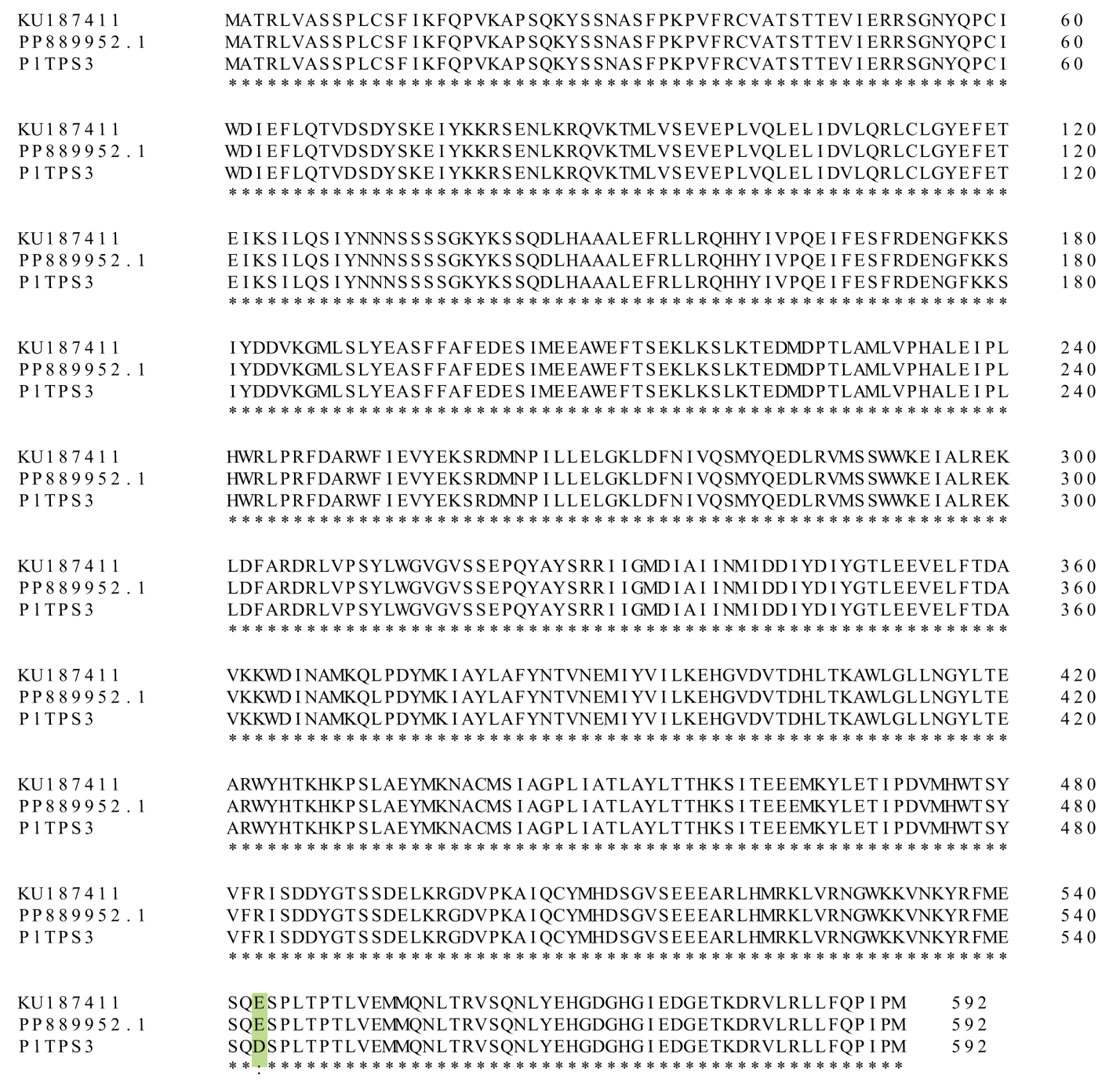


**Supplementary Figure S4.** Sequence alignment of KU187411, TPS3 from *P.lactiflora* ‘Zifengyu’ and PP889952.1 from *P. lactiflora* ‘Wu Hua Long Yu’. The residue polymorphism were highlighted with green backgrounds. Numbers represent positions of specific residues. *, identical amino acids, : or ·, similar amino acids.


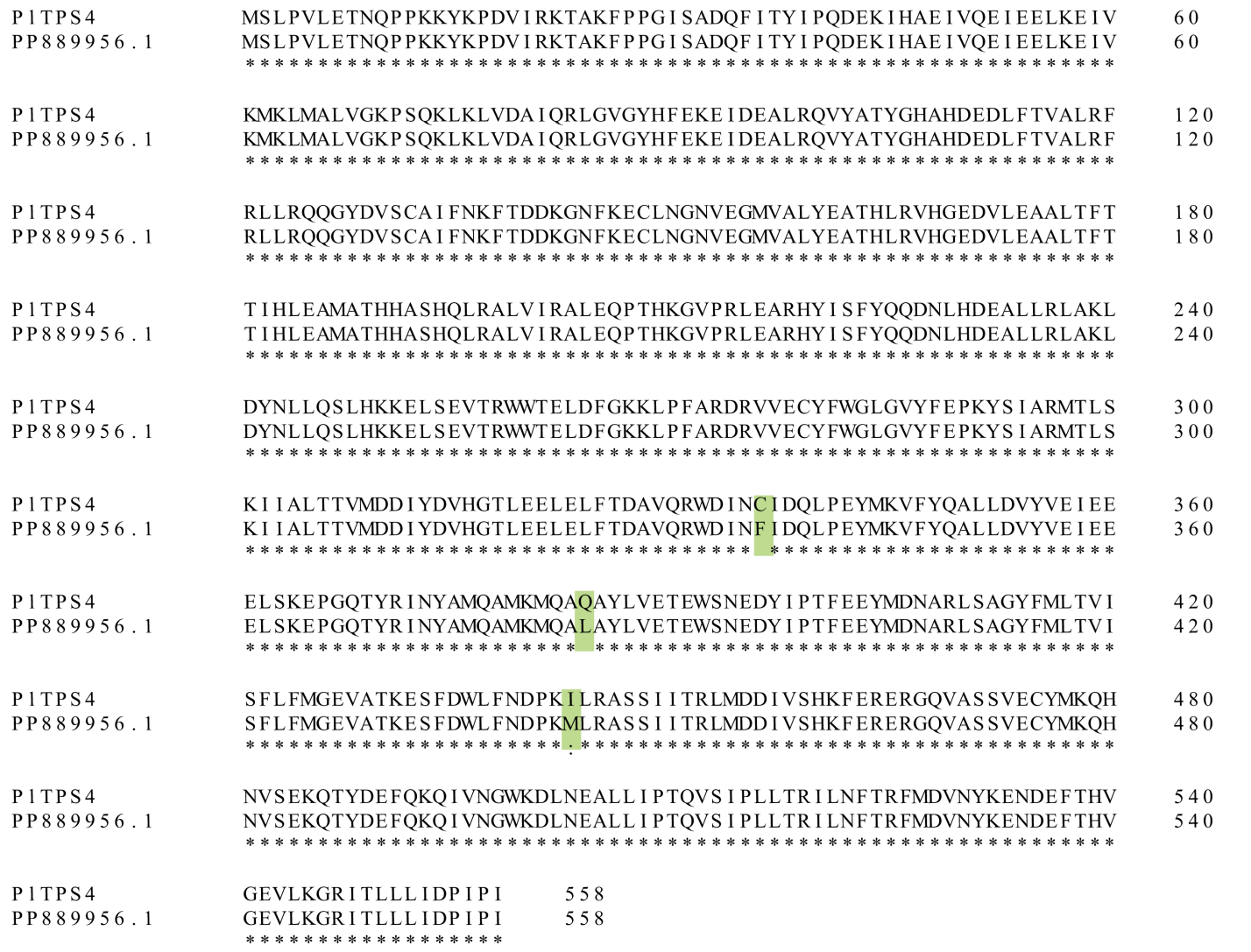


**Supplementary Figure S5.** Sequence alignment of TPS4 from *P. lactiflora* ‘Zifengyu’ and PP889956.1 from *P. lactiflora* ‘Wu Hua Long Yu’. The residue polymorphism were highlighted with green backgrounds. Numbers represent positions of specific residues. *, identical amino acids, : or ·, similar amino acids.


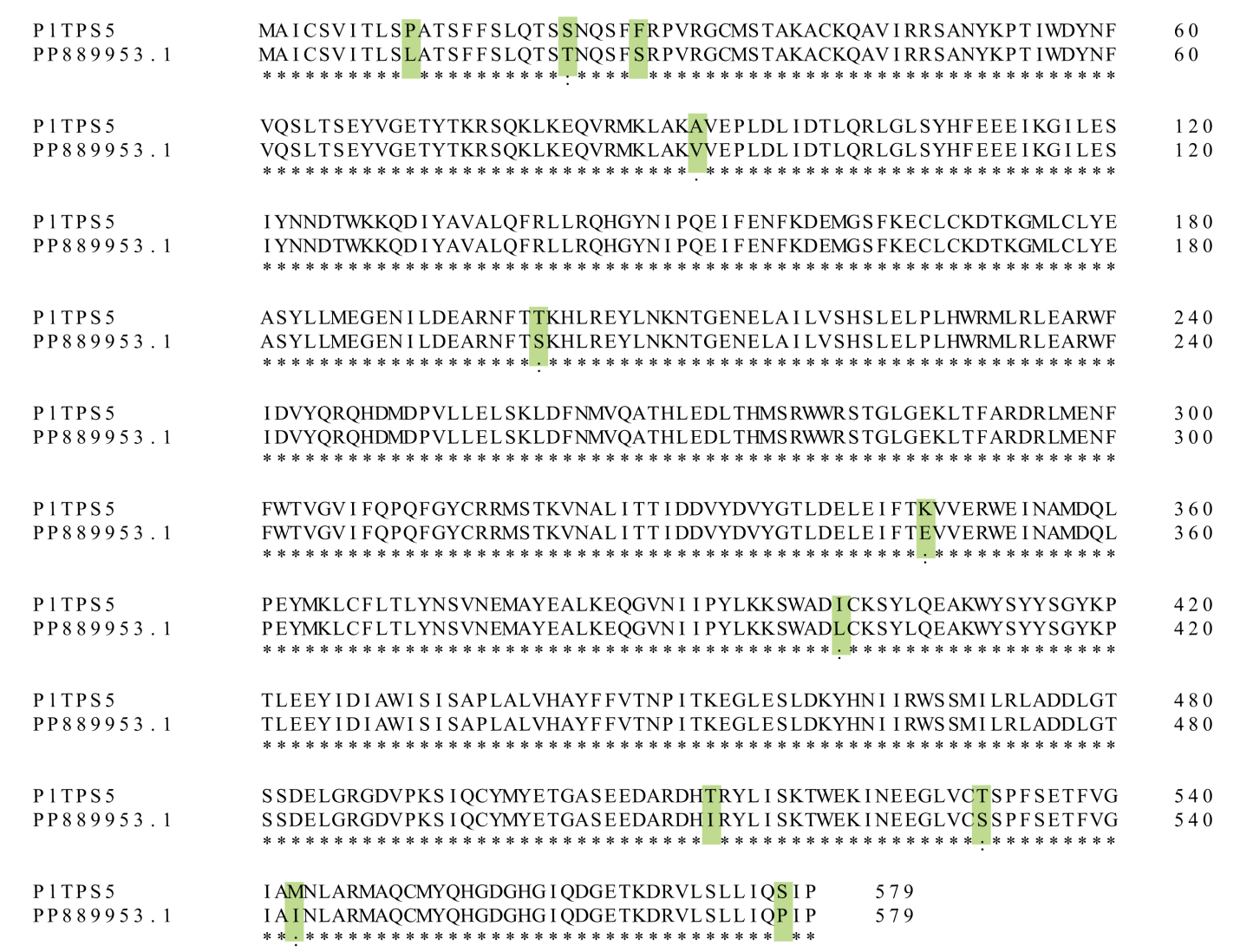


**Supplementary Figure S6.** Sequence alignment of TPS5 from *P. lactiflora* ‘Zifengyu’ and PP889953.1 from *P. lactiflora* ‘Wu Hua Long Yu’. The residue polymorphism were highlighted with green backgrounds. Numbers represent positions of specific residues. *, identical amino acids, : or ·, similar amino acids.


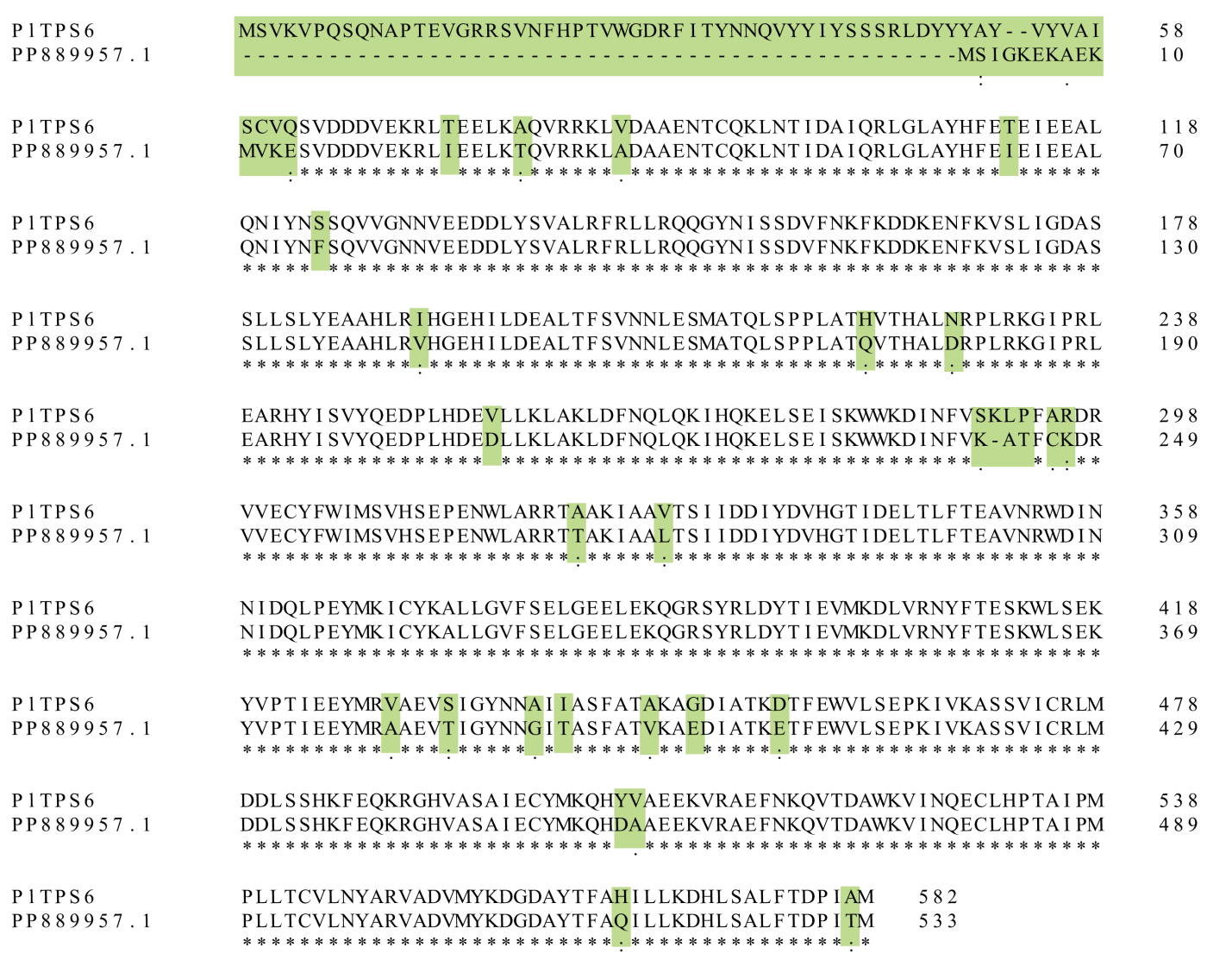


**Supplementary Figure S7.** Sequence alignment of TPS6 from *P. lactiflora* ‘Zifengyu’ and PP889957.1 from *P. lactiflora* ‘Wu Hua Long Yu’. The residue polymorphism were highlighted with green backgrounds. Numbers represent positions of specific residues. *, identical amino acids, : or ·, similar amino acids.


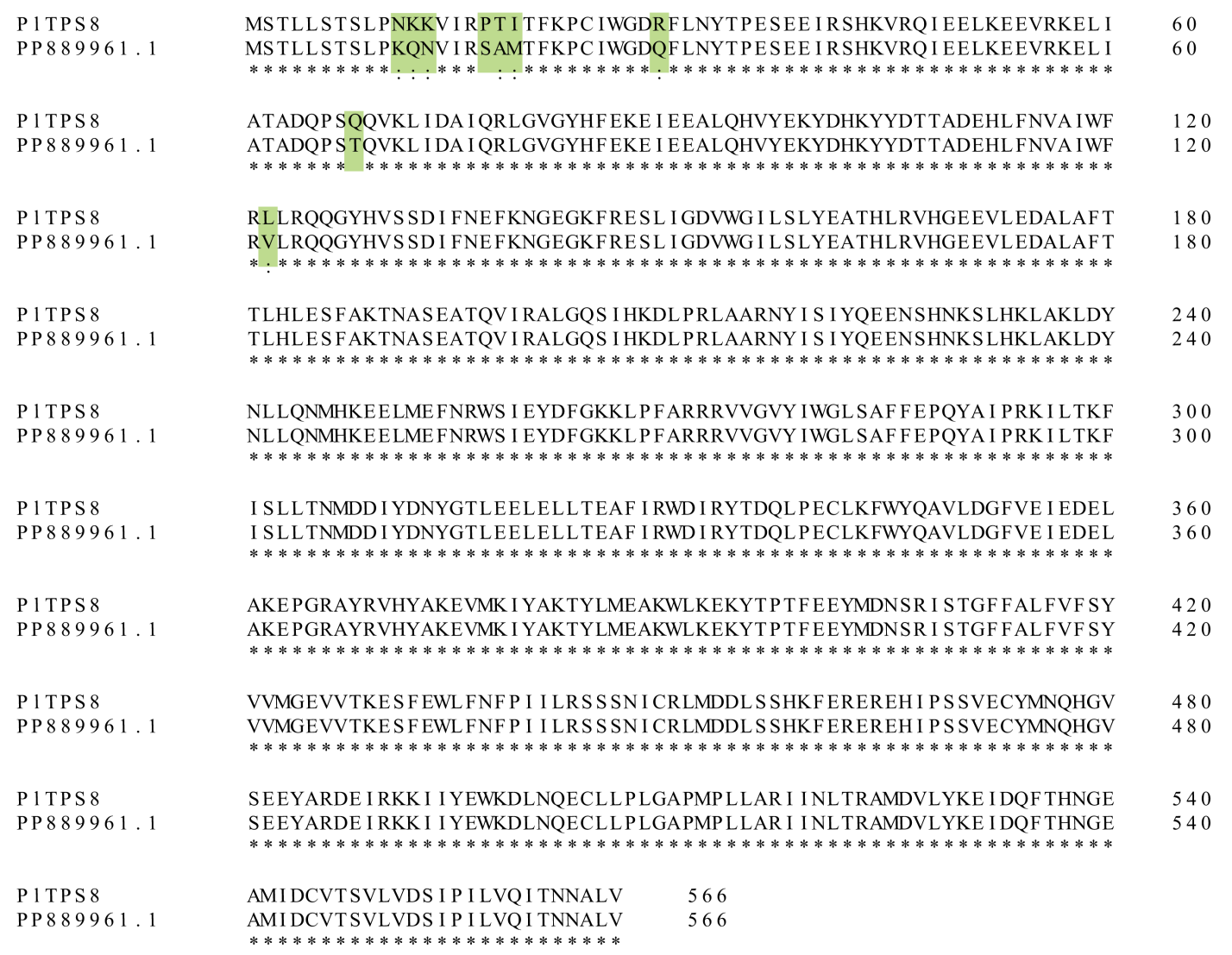


**Supplementary Figure S8.** Sequence alignment of TPS8 from *P. lactiflora* ‘Zifengyu’ and PP889961.1 from *P. lactiflora* ‘Wu Hua Long Yu’. The residue polymorphism were highlighted with green backgrounds. Numbers represent positions of specific residues. *, identical amino acids, : or ·, similar amino acids.


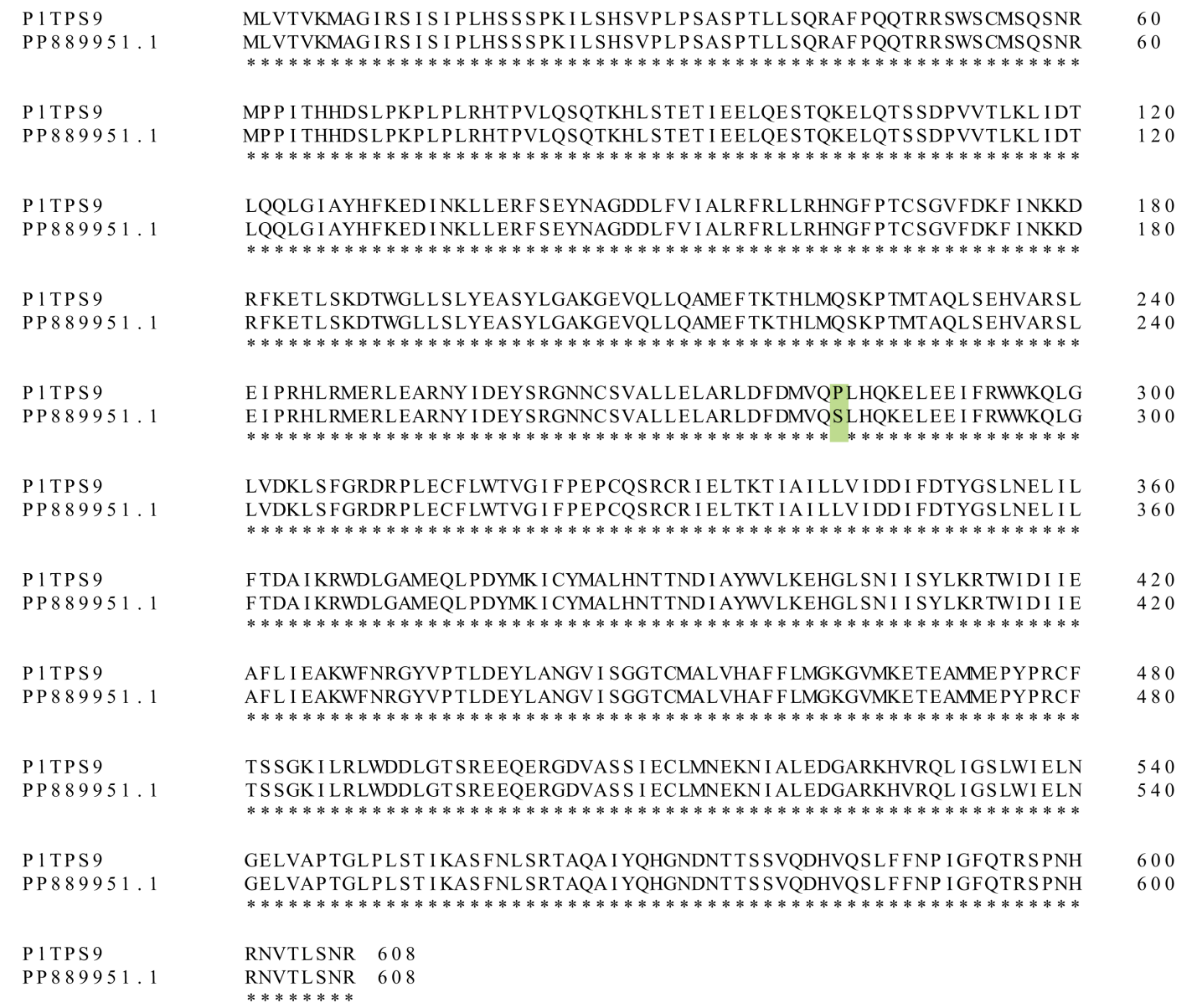


**Supplementary Figure S9.** Sequence alignment of TPS9 from *P. lactiflora* ‘Zifengyu’ and PP889951.1 from *P. lactiflora* ‘Wu Hua Long Yu’. The residue polymorphism were highlighted with green backgrounds. Numbers represent positions of specific residues. *, identical amino acids, : or ·, similar amino acids.


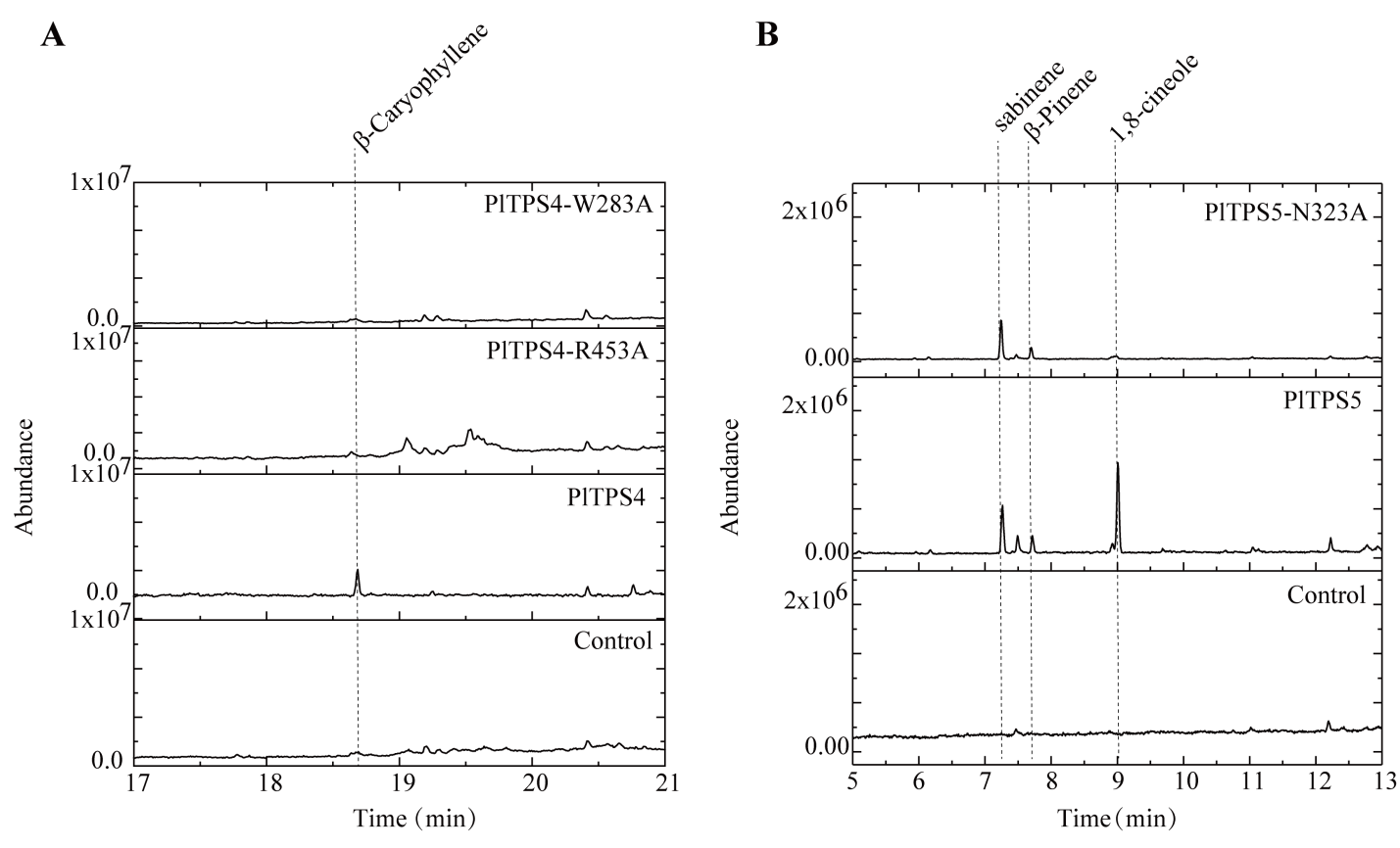


**Supplementary Figure S10.** Transient transformation of *TPS4* and *TPS5* genes and their variants in N. benthamiana. **A)** Heterologous transformation of *PlTPS4* and its mutants in N. benthamiana. **B)** Heterologous transformation of *PlTPS5* and its mutants in N. benthamiana. Volatiles were captured by HS-SPME and analyzed by GC–MS. Detailed quantification can be found in Supplementary Table S5. All data were calculated relative to β-caryophyllene (Bao et al., 2023) and the unit of content was ng/g fresh weight.


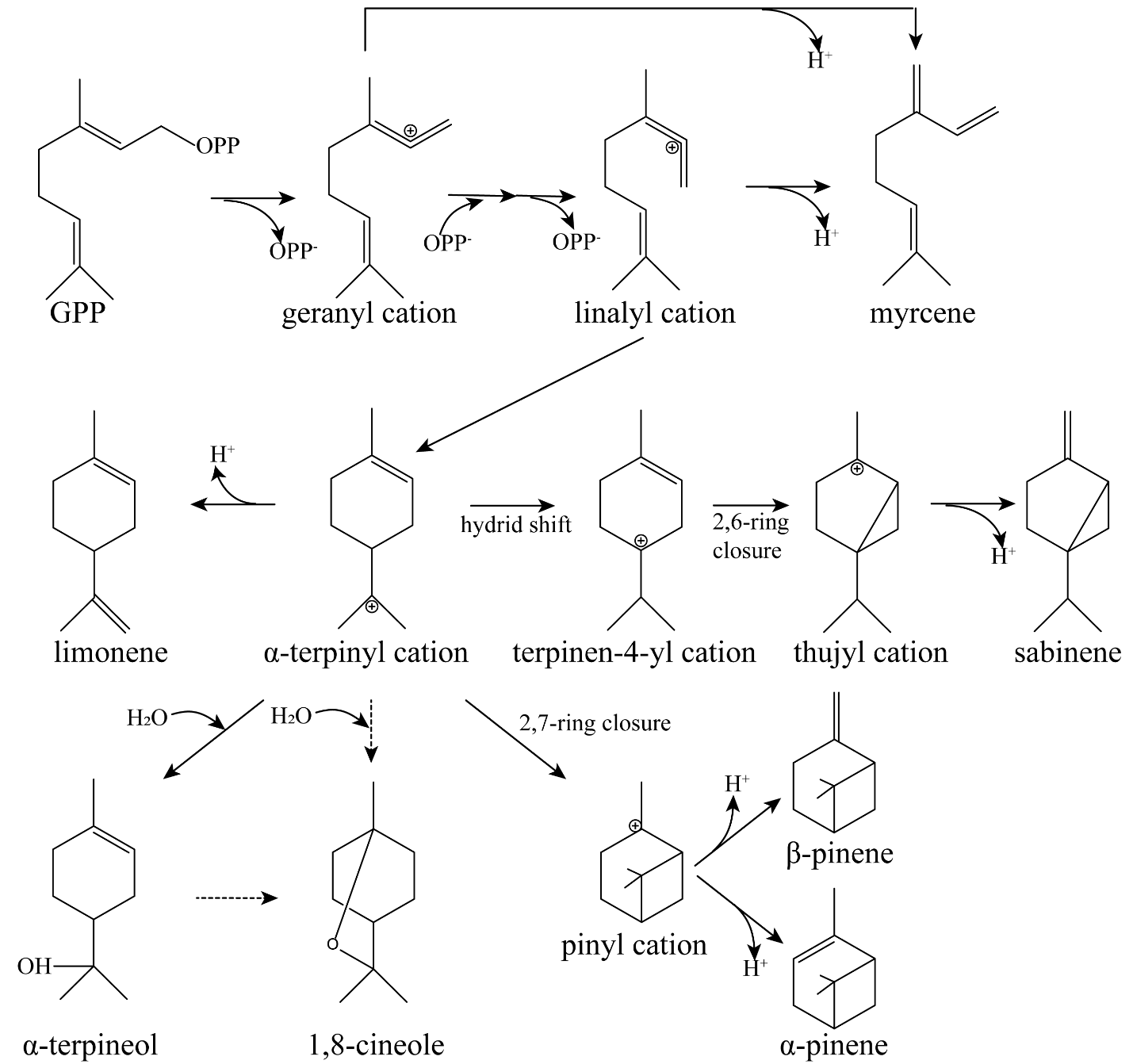


**Supplementary Figure S11.** Possible pathways for the synthesis of various monoterpenes. The substrate GPP is ionized by diphosphate elimination, resulting in the geranyl cation, linalyl cation and α-terpinyl cation (Piechulla et al., 2016).


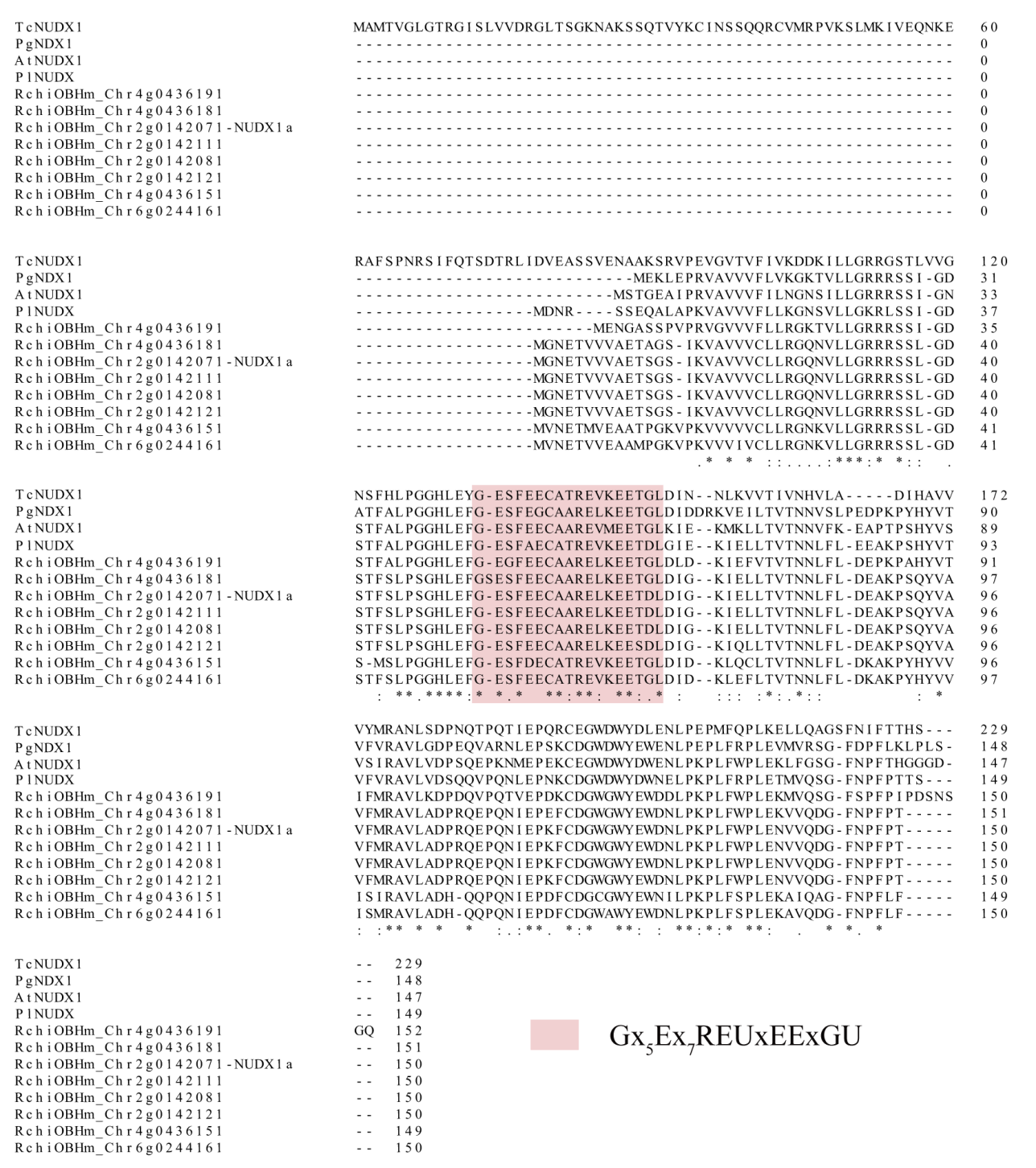


**Supplementary Figure S12.** Sequence alignment analysis of PlNUDX from *P. lactiflora* ‘Zifengyu’ and other plant species. The conserved Gx_5_Ex_7_REUxEExGU were highlighted with colored backgrounds. Numbers represent positions of specific residues. *, identical amino acids, : or ·, similar amino acids.
